# Sociolinguistic Variation in Mouthings in British Sign Language: A Corpus-Based Study

**DOI:** 10.1177/00238309221107002

**Published:** 2022-07-28

**Authors:** Heidi Proctor, Kearsy Cormier

**Affiliations:** Deafness Cognition and Language Research Centre, University College London, UK

**Keywords:** Sign language, deaf, BSL, mouthing, code-blending, code-switching, code-mixing, translanguaging, simultaneity, phonology, morphological complexity, lexicon, verbs, sociolinguistic variation, corpus linguistics

## Abstract

Mouth activity forms a key component of all sign languages. This can be divided into *mouthings*, which originate from words in the ambient spoken language, and *mouth gestures*, which do not. This study examines the relationship between the distribution of mouthings co-occurring with verb signs in British Sign Language (BSL) and various linguistic and social factors, using the BSL Corpus. We find considerable variation between participants and a lack of homogeneity in mouth actions with particular signs. This accords with previous theories that mouthings constitute code-blending between spoken and signed languages—similar to code-switching or code-mixing in spoken languages—rather than being a phonologically or lexically compulsory part of the sign. We also find a strong association between production of plain verbs (which are body-anchored and cannot be modified spatially) and increased mouthing. In addition, we observe significant effects of region (signers from the south of the United Kingdom mouth more than those from the north), gender (women mouth more than men), and age (signers aged 16–35 years produce fewer mouthings than older participants). We find no significant effect of language background (deaf vs. hearing family). Based on these findings, we argue that the multimodal, multilingual, and simultaneous nature of code-blending in sign languages fits well within the paradigm of translanguaging. We discuss implications of this for concepts of translanguaging, code-switching, code-mixing, and related phenomena, highlighting the need to consider not just modality and linguistic codes but also sequential versus simultaneous patterning.

## 1 Introduction

Although sign languages are often considered to be simply languages of the hands, it is clear that many other articulators play important roles, including elements of the face plus the head and torso. The mouth is a key component: indeed mouth activity has been found in virtually all sign languages investigated to date (Johnston et al., 2016). Mouth activity has been found to emerge early in the development of new sign languages. For example, Sandler (2012) found in her study of the newly emerging Al-Sayyid Bedouin Sign Language that mouth gestures appeared before the systematic use of facial gestures or of the body and before independent use of the non-dominant hand. The current study investigates the degree of variation in co-occurrence of mouthing with verb signs in British Sign Language (BSL) and linguistic and social factors that correlate with such mouthing, using the BSL Corpus (Schembri et al., 2014).

Mouth activity observed in signers can be divided into that which derives from the surrounding spoken language, known as *mouthing*,^1^ and that which does not, known as *mouth gesture*, as discussed in Boyes Braem and Sutton-Spence (2001). This study focuses on the distribution of mouthings in BSL, although mouth gestures (described briefly in section 1.2) are also identified in the data so as to distinguish them from other types (or lack) of mouth activity.

We argue that the use of mouthings at the same time as manual signs by BSL users can be viewed as a type of simultaneous production of two languages, English and BSL. This has parallels with switching between different spoken languages, known as *code-switching* or *code-mixing* (see, for example, Myers-Scotton, 1993), and also with translanguaging, which is the use of whatever linguistic repertoires (incorporating any modality) that are available to communicate effectively (see García, 2009). These concepts are introduced in section 1.1 . We go on to describe and categorize mouthings in section 1.2, then discuss their possible linguistic status in section 1.3. Next, we discuss factors that can affect the rate of mouthing in section 1.4, and we conclude the introduction by summarizing past research exploring the rate of mouthing in BSL and other sign languages in section 1.5.

### 1.1 Code-switching and translanguaging in spoken languages

Code-switching in spoken languages has generally been studied in situations where all conversational partners are fluent in the same set of languages (MacSwan, 2014). Various models of code-switching in spoken languages have been proposed, including Myers-Scotton’s (1993) suggestion that in any utterance involving code-switching among bilinguals, there is a *matrix* language into which elements of an *embedded* language are inserted. Other researchers have put forward constraints relating to the points within an utterance at which a code switch is permitted, such as the Equivalence Constraint (Poplack, 1980). This states that code switches tend to only occur where the result matches the word order of both of the languages in question. In contrast, MacSwan (2000) suggests a constraint-free approach, providing counterexamples to the proposed constraints and instead proposing that code-switching is governed only by the rules of each language’s syntax and that no overarching rules discussing code-switching itself are required to describe the process.

More recent research on bilingual/multilingual practices has broadened the remit beyond code-switching to translanguaging. Translanguaging is the use of multiple linguistic repertoires: this may include switching or mixing of any number of languages—spoken and/or signed—as well as gesture, intonation, and writing (García, 2009; Hodge & Goswell, 2021; Kusters et al., 2017). There is some debate about whether the traditional notion of code-switching is included within translanguaging and whether code-switching can/should be studied separately at all (Chan, 2021; Wei, 2018a, 2018b). Auer (under review) argues that it is important to distinguish between code-switching and other bilingual/multilingual practices that involve mixing or borrowing or merging, that code-switching implies the existence of clearly distinct “codes” (i.e., named languages) whereas mixing, borrowing, and merging (what he considers to be translanguaging) do not.

As with other linguistic phenomena, there has been sociolinguistic variation reported in code-switching (e.g., Myers-Scotton, 1993; Poplack, 2018). Translanguaging is inherently highly individualized and variable (Auer, under review; García, 2009; Kusters et al., 2017).

In sign languages, use of mouthing with manual signs has been considered similar to code-switching in spoken languages, but because it involves simultaneous rather than sequential patterning it has been referred to as “code-blending” (see section 1.3). Code-blending is considered typologically unique to sign languages (Bank et al., 2011). We propose that code-blending is simply one type of translanguaging, which blends elements of both a signed and a spoken language. We return to this issue in section 5.8.

In the following section, we provide some background about mouthings and mouth gestures in signed languages.

### 1.2 Introduction to mouthings and mouth gestures

Mouthings have a variety of uses in sign languages. In many cases, they provide disambiguation between polysemous senses of a sign, that is, where one manual sign has several related meanings. For example, in BSL and also Australian Sign Language (Auslan), the manual sign road (https://bslsignbank.ucl.ac.uk/dictionary/words/road-2.html) can mean “road” or “method” or “way” depending on context and/or mouthing (Johnston & Schembri, 2007).

In other cases, mouthings can disambiguate between manual homonyms in different semantic fields, such as aunt (http://bslsignbank.ucl.ac.uk/dictionary/words/aunt-1.html) and battery (http://bslsignbank.ucl.ac.uk/dictionary/words/battery-1.html) in BSL. Similarly they can be used to differentiate between fingerspelled^2^ abbreviations of English words that use the same letter or sequence of letters (Sutton-Spence, 1994), such as the BSL signs garage (http://bslsignbank.ucl.ac.uk/dictionary/words/garage-1.html) and geography (https://bslsignbank.ucl.ac.uk/dictionary/words/geography-1.html), which both involve a repetition of the fingerspelled letter -g-.

In addition, mouthings may aid a signer who is not familiar with a particular sign to comprehend it: see Stamp (2016), for BSL, in the context of regional signs. According to Boyes Braem (2001), this is also very common in Swiss German Sign Language (DSGS) because, as is the case for many sign languages, it has no standard form. This means that conversational partners often do not share the same lexicon of manual signs, so they use mouthings to exploit the fact that they do share some knowledge of the same ambient spoken language.

Mouthings may add semantic information: for example in Sign Language of the Netherlands (NGT) one may mouth the Dutch word for “bread” at the same time as making the manual sign eat to produce the meaning “eat bread,” as noted by Crasborn et al. (2008). Similarly, mouthings can modify the meaning of adjectives or adverbs. For example, they can add intensity as in the mouthing of the German word for “very” along with the manual sign good in DSGS (Boyes Braem, 2001). Boyes Braem (2001) also notes that mouthings can occur without an accompanying manual sign in DSGS, either because of a lexical gap in the sign language or because of a gap in the signer’s knowledge of that language.

Finally, mouthings can be redundant in that they convey the same information as is provided by the hands (Boyes Braem, 2001; Ebbinghaus & Hessmann, 2001). As noted in Hohenberger and Happ (2001), there is a tradeoff in all languages, spoken and signed, between redundancy on one hand, which aids reliable communication, and economy on the other, which increases the speed of information exchange. It may be that in languages such as sign languages that have no written form and that are therefore more ephemeral, additional redundancy is encoded “in order to fix somehow the content of the utterance” (Fontana, 2008, p. 12).

Mouthings are distinguished from mouth gestures, which are instances of mouth activity that do not derive from the surrounding spoken language. As noted in section 1, we do not discuss mouth gestures in detail in this paper since our focus is on mouthings. Crasborn et al. (2008) provide a typology of mouth gestures as part of their investigation of the distribution of mouthings and mouth gestures in three sign languages. Whole-face mouth gestures, which are further subdivided and reviewed in Johnston et al. (2016), are not included in Crasborn et al.’s discussions since these are part of wider facial activity and generally relate to emotion. Boyes Braem and Sutton-Spence (2001) also suggest that these should be treated separately.

The distinction between mouthings and mouth gestures is not always clear-cut, as pointed out by Vogt-Svendsen (2001) and by Schermer (2001). The viewer’s previous experience will affect whether or not they perceive a given mouth pattern as derived from a word in the ambient language. For example, Lewin and Schembri (2011) note several tokens of tongue protrusion with the BSL sign nothing in their dataset which were ambiguous between the semantically empty mouth gesture [θ] versus [θ] as a reduced mouthing of “nothing.” Also, Siu (2007), in her investigation of mouth activity in Hong Kong Sign Language (HKSL), studied a deaf native signer. She recorded this informant’s opinions as to whether her own mouth actions were mouthings or mouth gestures: these did not always coincide with those of another deaf native HKSL user who acted as a reviewer.

### 1.3 Linguistic status of mouthings

Deaf communities typically exist in the context of larger hearing communities and in these cases, there is an inevitable influence of the ambient spoken language upon a given sign language. However, the degree to which mouthing occurs in a sign language varies considerably: for example, de Vos and Zeshan (2012) report that Kata Kolok (a village sign language of Bali) has virtually no mouthing. In many sign languages, however, mouthing is common. Indeed, it has been observed for many sign languages that mouthing persists even when native signers are conversing with one another with no hearing people present, as noted by Johnston et al. (2016) for Auslan.

The linguistic status of mouthings within sign languages has long been a topic of debate: see, for example, chapters within Boyes Braem and Sutton-Spence (2001). Some chapters in this volume, such as Sutton-Spence and Day (2001), Boyes Braem (2001) and Vogt-Svendsen (2001), conclude that although they originated as borrowings from the surrounding spoken language, some mouthings have become a compulsory, inherent part of the phonological/lexical specification of the sign they accompany. For example, Sutton-Spence and Day (2001) analyzed 7,992 manual sign tokens of BSL and found that certain plain verbs^3^ such as know (https://bslsignbank.ucl.ac.uk/dictionary/words/know-1.html), (which can mean “know,” “knowledge,” “knowledgeable,” “aware”) were always produced with the same mouthing (“know” in this case) based on seven manual tokens of this sign.

However, more recent studies (e.g., Bank, 2015; Ebbinghaus & Hessmann, 2001; Giustolisi et al., 2017; Hohenberger & Happ, 2001) have concluded that mouthings are instead independent meaningful units that enhance the overall meaning of the utterance. They hold that the signer can (subconsciously) select what mouth activity (including mouthing, mouth gesture, or no mouth activity) to produce with each manual sign. When mouthing is selected, this is a process many consider to be an example of *code-blending*. This term was originally coined by Emmorey et al. (2005) as an extension to the concept of code-switching in spoken languages discussed in section 1.1. Emmorey et al. defined code-blending as the simultaneous production of spoken words and manual signs by hearing bimodal bilingual individuals, that is, hearing individuals from deaf signing families who are native users of both a signed and a spoken language. Bank et al. (2011) extended the concept of code-blending to include the nonvoiced mouthing of spoken words with manual signs by deaf signers, since any vocalizations that may be produced are irrelevant in the context of a deaf conversational partner. In this extended notion of code-blending, mouthings are considered to be (as with vocalized words) simultaneous expression of words or parts of words from a different language.

As evidence to support the notion of mouthings as code-blending in sign languages, researchers have observed that mouthings are not obligatory and that individuals can produce widely differing amounts of mouthings (see Johnston et al., 2016, for Auslan). Similarly, Bank et al. (2011) observed considerable variation in whether mouthings of spoken Dutch are produced with particular signs in NGT. They found that in the 20 most frequent signs in their corpus of NGT, the rate of mouthing varied between 29% and 100%. In their sample, only 3 of the 20 signs were accompanied by mouthing 100% of the time. In addition, Bank et al. found that their participants were not consistent in their temporal reduction of Dutch words when mouthing, or in how these mouthings are temporally aligned to manual signs. A code-blending analysis is also supported by psycholinguistic evidence. Vinson et al. (2010) asked native signers to produce a BSL sign for each of a series of pictures and of written words, as quickly as possible. The researchers analyzed the errors that were produced, both on the mouth and on the hands. They theorized that if there was a single semantic representation containing both the manual sign and corresponding mouthing in the signer’s mental lexicon, then any semantic errors would manifest themselves on both the hands and the mouth. In fact, different patterns of errors were observed, suggesting that the mouthing and the manual sign are represented separately in the brain. Vinson et al. argue that this supports a code-blending analysis because it implies that when producing utterances, signers retrieve mouthings and manual signs from these distinct mental representations, from two different languages.

### 1.4 Factors affecting rates of mouthing

Various factors have been found to affect the rate of mouthing in sign languages. These include the morphological complexity of the corresponding manual sign and the type of situation in which the communication occurs (i.e., text-type). In addition, social factors such as the region where the signer lives, their gender, age, and whether they grew up with any deaf relatives (known as their language background) may all play a part. These will be discussed in turn, with reference to studies of different sign languages, some of which considered several of the factors.

#### 1.4.1 Verb type

Mouth activity is not found uniformly with all the manual signs in a sign language. While mouthings occur frequently with noun signs in many sign languages, their use accompanying verbs has been found to vary more. For example, Johnston et al. (2016) studied mouth activity in Auslan, a language closely related to BSL. They annotated 17,002 manual tokens from 50 extracts from the Auslan Corpus, consisting of 25 monologue narrative retellings of Aesop’s fables, 15 elicited picture descriptions, and 10 dialogues that were a mixture of free conversation and answers to interview questions. They found that in their sample, verbs were mouthed 28% of the time and nouns were mouthed 78% of the time. Other studies, such as Bank et al. (2015), Johnston et al. (2015), Rentelis (2011), and Boyes Braem and Sutton-Spence (2001) similarly found a much higher frequency of mouthing with nouns compared with verbs. This may be at least in part because nouns are generally less morphologically complex than verbs in sign languages (cf. Meir, 2012; Padden, 1983).

However, even within the grammatical category of verbs, not all verb types in sign languages are morphologically complex. In fact, verb types exhibit varying degrees of morphological complexity. Across many sign languages, verbs have been categorized as plain, indicating, or depicting (Liddell, 2003). Plain verbs are those that are body-anchored and where the hands cannot be modified spatially. Indicating verbs can be directed spatially at or toward places, entities, or directions (Liddell, 2003).^4^ Depicting verbs, also known as classifier verbs or classifier constructions, portray aspects of their meaning including features of the object that they are describing. Since plain verbs cannot be modified spatially via path movement of the hands, they are less morphologically complex than indicating or depicting verbs: they do not encode concepts such as the subject and object or source and goal of the verb within the sign. Hohenberger and Happ (2001) observe that in German Sign Language (DGS) it is not possible to mouth the full set of translation equivalents included in morphologically complex signs such as indicating and depicting verbs. Therefore, signers either produce no mouthing at all with these signs, or they mouth just the stem of the verb. Overall, this reduces the frequency of mouthings on these more morphologically complex signs.

Differences in mouthing across verb type have also been found in BSL and American Sign Language (ASL). In the studies referenced above, Sutton-Spence and Day (2001) found that in BSL, only 23% of verbs associated with mouthings were morphologically complex, and Nadolske and Rosenstock (2007) observed that in ASL, mouthings occurred on 53% of plain verbs, 38% of indicating verbs, and 7% of classifier verbs. In addition, many of the other studies reported in Boyes Braem and Sutton-Spence (2001) found a similar effect. For example, Vogt-Svendsen (2001) observed that mouthings occurred more on unmodified verbs than on indicating or depicting verbs in Norwegian Sign Language.

Johnston et al. (2016) also noted that previous research found more mouthing co-occurring with plain verbs compared with indicating or depicting verbs. However, their own study of mouth activity in Auslan did not find this: instead, they found that indicating verbs patterned differently than had previously been observed, in that their rate of mouthing (36%) was similar to—if anything, actually slightly higher than—plain verbs (34%). Both of these were much higher than for depicting verbs (3%). As noted by Cormier et al. (2012), it may be that mouthing occurs more on fully lexical signs (including plain and indicating verbs) compared with depicting verbs which are only partly lexical.

Overall, a pattern is clear across the range of languages discussed in this section, with higher rates of mouthing on morphologically simpler signs such as nouns, compared with morphologically more complex signs such as depicting signs.

#### 1.4.2 Text-type

Past research has found that the rate of mouthing differs by text-type. Sutton-Spence and Day (2001) analyzed BSL in texts in what they termed “information register” (formal interviews, television news interpreting, and lectures) and in “narrative register” (recounting a dramatic news story, telling personal narratives, and retelling a fantasy story). They found that mouthing occurred on 77% of signs in the information register and 50%^5^ of signs in the narrative register. However, it is possible that these results could be skewed by the fact that the study involved quite different numbers of tokens and participants for each task, ranging from a single television news interpreter providing 824 tokens to 12 participants retelling the fantasy story, producing 2,210 signs between them.

Looking at other sign languages, Johnston et al. (2016) observed that there were significantly fewer mouthings in Auslan associated with narrative retellings (20.3% of tokens) compared with dialogues (68.6% of tokens). Although there was considerable variation between individuals in their study, almost all of the people who participated in tasks using more than one text-type showed more mouthings with dialogues than narratives, suggesting that the text-type difference is real. Similarly, Nadolske and Rosenstock (2007) studied 5,785 manual tokens in ASL from three text-types: storytelling, conversation, and formal lecture. They also noted a lower occurrence of mouthings with narratives, observing their occurrence with 60% of signs in the conversation and formal lectures, but only with 42% of signs for the narratives. Furthermore, Van De Sande and Crasborn (2009) found a significant difference between narrative and conversation text-types in a study of narrative retellings compared with spontaneous discussions in a sample of 12 signers taken from their corpus of NGT: 47% of mouth activity consisted of mouthings for the narratives, compared with 78% of mouth activity being mouthings in the conversations.

However, not all studies have found a difference based on text-type. Schermer (2001) compared the rate of mouthing of up to six participants undertaking different tasks in NGT. Out of 4,279 tokens, she found that mouthing occurred with similar percentages of manual signs for all text-types, as follows: retelling two written stories (55.4% and 62.4%); retelling a picture story (46.6%); and free conversation with another deaf participant (51.3%). She suggests that this indicates that the Dutch words in the written stories and the presence of a deaf conversation partner do not greatly influence the rate of mouthing in NGT.

In sum, previous research has shown mouthing to be generally found less often in narrative compared with conversation text-types.

#### 1.4.3 Social factors

Social factors such as gender, age, ethnicity, region, and social class are known to influence the general linguistic production of individuals: this is true for both spoken and signed languages (Lucas, 2001). The influence of social factors on mouthings is discussed below. Cross-linguistically, not all studies find that social factors are significant predictors. For example, Johnston et al. (2016) did not find age, gender, language background, or region to be significant predictors of mouthing in Auslan.

##### 1.4.3.1 Region

Regional variation has been identified in BSL particularly in the lexicon (e.g., Stamp et al., 2014) and also in fingerspelling (Brown & Cormier, 2017; Sutton-Spence et al., 1990). There has been less research on regional variation in mouthing in BSL. Rentelis (2011) notes that the “folk wisdom” among the British deaf community is that Scots use mouthing less than southern English signers. He investigated this using the BSL Corpus (see section 3.1), studying 250 verb tokens each from London and Glasgow, and found that there was indeed a significant difference in rate of mouthing in these two regions. Rentelis did not find a similar result for mouthings accompanying nouns. His study is relatively small in scale, and he does not provide details about how he selected which tokens to include or how he identified their grammatical category: this is not straightforward in sign languages as discussed in section 3.2.1.

##### 1.4.3.2 Gender

Rentelis’ (2011) study also found that women produced significantly more mouthings accompanying verb tokens compared with men in the BSL Corpus. In countries such as Ireland with a different education policy whereby gender-segregation of deaf children has been the norm, gender differences in mouthing have also been observed. For example, Mohr (2012), also found a higher rate of mouthing in Irish Sign Language among women than men: she suggests that their different educational experience is the reason for the variation. However, in nations such as the Netherlands and Australia, where there has been no such educational segregation, no significant difference between males and females is found in mouthing (Bank, 2015; Johnston et al., 2016). Rentelis suggests that the gender difference found in BSL may actually be an effect of social class, citing Ladd (2003). We return to this issue in section 5.4.

##### 1.4.3.3 Age

An individual’s experience of English and BSL during their education is likely to affect their signing throughout their life. This will include their mouthing (Sutton-Spence & Day, 2001). In Stamp’s (2013) study, she notes that the age of a BSL signer can be used as a proxy for their education background, because of national changes in education policy over time. As explained in Schembri et al. (2013), despite the suppression of sign language in the classroom during most of the 20th century in the United Kingdom, its use continued covertly. During the first half of the century deaf children were generally taught in specialized residential schools using a great deal of fingerspelling and speech-reading. After the Second World War, there was a greater emphasis on speaking and using residual hearing. Then later, schools for deaf children began to close and pupils were instead educated in a mainstream setting. Sign-supported English (SSE)^6^ was introduced and individuals were offered improved hearing aids as technology advanced. Finally, those starting their education from approximately 1980 onwards were generally taught in mainstream schools with BSL interpretation. Some, however, were educated using BSL in schools that adopted a bilingual English/BSL approach.

Previous research has investigated the correlation between age and rate of mouthing in BSL. For example, Sutton-Spence and Day’s (2001) research compared signers above and below the age of 40 at the time of filming, so born before/after approximately 1957. They did not find a significant difference, but perhaps this is not surprising since both these groups will have experienced a similar oral education in schools for the deaf. However, in another part of their study, they compared signers aged above and below 30 years (born before/after 1967). This is approximately the age at which the older group would have been educated in schools for the deaf and the younger group in mainstream schools. The younger group produced more mouthings, although the differences were small. Rentelis (2011) did not find an effect of age on the degree of mouthings with verbs when examining the BSL Corpus, which was filmed between 2008 and 2010. He categorized people into “young” (born between approximately 1974–1991), “middle” (born between around 1959 and 1973) and “old” (born before 1959). His middle/old distinction is comparable to the above/below age 40 cutoff used by Sutton-Spence and Day, and his findings are the same (i.e., no significant difference between those above/below this cutoff). However we cannot compare Sutton–Spence and Day’s above/below age 30 result to Rentelis as he did not make a corresponding age split.

##### 1.4.3.4 Language background

A further social factor is *language background*, that is, whether participants grew up with any signing close relatives or other caregivers from whom they acquired a sign language. This is relevant because only 5%–10% of deaf people acquire sign language from signing family members (Mitchell & Karchmer, 2004; Schembri et al., 2013), and the presence of signing relatives affects an individual’s exposure to BSL during their critical period for language acquisition (Mayberry, 2007). Sutton-Spence and Day (2001) state that Day (1995) found that signers without deaf relatives used BSL with more English influence. However, their own research on BSL found no significant difference in the use of either mouthings or mouth gestures based on language background. Similarly, the other major studies on mouth activity in sign languages, including Johnston et al. (2016) and Bank et al. (2015), as well as Rentelis (2011), looked for but did not find variation based on the language background of participants.

### 1.5 Cross-linguistic variation in mouthing proportions

Key studies on the rate of mouth activity in various sign languages, including those discussed above, are summarized in Table 1. Due to the different methodologies employed in each piece of research, direct comparison is not possible: some studies report the split of all mouth activity (excluding no mouth activity) into mouthing and mouth gesture (Table 1, columns 3 and 4),^7^ whereas others report the percentage of manual signing that is accompanied by mouthing, mouth gesture, or no mouth activity (Table 1, columns 5–7). Nonetheless, it is evident that widely different results have been obtained, even within the same language. One reason for this may be differences in text-type: for example, the earliest study of NGT (Crasborn et al., 2008) found that only 47% of mouth activity was mouthing, whereas Bank (2015) found a rate of 85% in the same language. The first of these used a story retelling task, whereas the second used conversation.

**Table 1.** Cross-Linguistic Summary of Percentage of Mouth Activity.

Language	Study	% of mouth activity (excluding no mouth activity)		% of mouth activity with manual signs		
		Mouthing	Mouth gesture	Mouthing	Mouth gesture	No mouth activity
ASL	Nadolske and Rosenstock (2007)	–	–	42–60	–	24–34
Auslan	Johnston et al. (2016)	74	26	57	20	23
BSL	Crasborn et al. (2008)	64	36	36	20	29
BSL	Sutton-Spence and Day (2001)	80	20	69	17	14
DGS	Ebbinghaus and Hessmann (2001)	–	–	50	–	–
HKSL	Siu (2007)	54	46	–	–	–
LIS	Ajello et al. (2001)	89	11	49	6	45
NGT	Crasborn et al. (2008)	47	53	26	29	35
NGT	Bank (2015) ^a^	85	15	61	10	27
NGT	Bank et al. (2015)	74	–	–	–	–
SSL	Crasborn et al. (2008)	68	32	51	24	10

*Note*. ASL: American Sign Language; BSL: British Sign Language; DGS: German Sign Language; HKSL: Hong Kong Sign Language; LIS: Italian Sign Language; NGT: Sign Language of the Netherlands; SSL: Swedish Sign Language.

Dashes indicate where data are not available. Figures for “% of mouth activity with manual signs” which do not total 100% are because some mouth actions in these studies could not be categorized.

a Whole-face mouth gestures are counted as “no/other mouth activity” in the current study (see section 3.2.2), so they are generally not included in this table. However, Bank (2015) does not distinguish them from other types of mouth gesture, so they are included in this row under “Mouth gesture.”

## 2 Research questions and hypotheses

In this study we investigate the amount of variation in the rate of mouthing between participants, and with particular signs. In addition, we consider the correlation between the rate of mouthing and the following linguistic and social factors: verb type, region, gender, age, and language background. As noted in section 1.4.1, in many studies across many sign languages, mouthings have been noted to occur frequently with nouns but their use alongside verbs has been found to vary. This may be because of the different degrees of morphological complexity in verbs; by contrast, nouns tend to exhibit less morphological complexity. Because we are focusing on rate of mouthing rather than, for example, what word or part of a word is mouthed, we focus on verb signs in this study.

Our first set of research questions ask: What is the degree of variation in the rate of mouthing on verb signs between our participants? How much variation is observed in the rate of mouthing on particular verb signs? We noted in section 1.1 that variation in spoken language code-switching and in translanguaging has been observed, and in section 1.3 that there is variation in mouth activity both between participants and for given signs, in Auslan and in NGT. This question has not yet been explored in BSL.

We then consider: What is the correlation between the rate of mouthing and the type of the corresponding manual verb sign? As discussed in section 1.4.1 above, previous research in many sign languages has indicated that there is a relationship between these factors, but this study is the first to investigate this using a large dataset of BSL. Plain verbs are assumed to be the most morphologically simple, so our hypothesis is that the rate of mouthing on plain verbs is higher than on non-plain verbs.

In terms of social factors—region, gender, age, and language background—we take the work of Rentelis (2011) as a starting point. As for region, Rentelis analyzed verb tokens produced by 25 participants in each of London and Glasgow (10 verb tokens for each of the 25 participants in each city, for a total of 500 verb tokens). In the current study, we extend this to include individuals from Bristol as well as London in the south of the United Kingdom, and Belfast as well as Glasgow in the north. This provides a more varied sample in terms of region, with two cities in the southern part of the United Kingdom and two cities in the north, which enables us to further test the “folk wisdom” within the British Deaf community that signers in the north of the United Kingdom produce less mouthings than those in the south. In addition, our sample size is larger than Rentelis,’ with twice as many cities and thus participants (25 per city, for a total of 100). For details of our methodology regarding the number of verb tokens included, see section 3.2. Our research questions examine the effect of region, gender, age, and language background on the rate of mouthings produced with verbs. We expect to find, as Rentelis (2011) did, a lower rate of mouthing accompanying verbs in signers from the north of the United Kingdom compared with those from the south. Similarly, in common with Rentelis’s findings, we expect to find that women produce more mouthings on verb signs than men.

Previous studies on the relationship between age and rate of mouthing in BSL have obtained mixed results: Sutton-Spence and Day (2001) found a correlation whereas Rentelis (2011) did not. We hypothesize that people aged 36–50 years at the time of corpus data collection use more mouthings than those of other age groups. This is because these individuals were likely to have been educated in a mainstream environment, with fewer deaf peers at school. This would entail reduced BSL input: instead, they would have likely interacted with a Communication Support Worker using SSE. Therefore, English will have exerted a greater influence upon their signing, and they will use more mouthings accompanying verbs than the rest of the population. By contrast, signers in all the other age groups experienced mixed influences upon their mouthing, some factors serving to increase it and some to decrease it. Signers in the two oldest age groups in the BSL Corpus (aged 51 and over) were generally educated orally in residential schools for the deaf. The oral education might tend to increase their rate of mouthing, whereas their centralized education promoted the use of BSL informally, which may have reduced their mouthing. The youngest signers (aged 16–35 years) mostly attended mainstream schools, which might tend to increase mouthing because of their contact with hearing classmates, but the use of BSL in the classroom might reduce the influence of English and therefore reduce their mouthing.

No effect of language background has been found in previous studies of mouth activity in BSL and other sign languages as discussed in Section 1.4.3.4. Therefore, we predict that there will be no difference between the rate of mouthing accompanying verbs produced by signers who grew up with and without deaf relatives.

## 3 Method

### 3.1 Data

The BSL Corpus (Schembri et al., 2013) formed the key data source for this project. During its development, participants were chosen using non-random quota-based techniques with the aim of matching the proportions of age, gender, language background, region, and ethnicity in the overall deaf community in the United Kingdom. All participants were deaf, and 95% said that they had been signing since at least the age of 7, with the remainder learning before age 12. Participants were selected to match the changes in education policy in the United Kingdom described in section 1.4.3.3, with approximately equal numbers of people recruited in each of the following age brackets: 65 years and older, 51–64 years, 36–50 years, and 35 years and younger. They were filmed in pairs performing a variety of linguistic tasks, including recounting a personal narrative and engaging in spontaneous free conversation. For personal narratives, participants were asked ahead of time to think of a 5-minute personal story (e.g., funny, sad, intriguing): some participants did this while others forgot, and their narratives ended up being spontaneous.

We selected a subset of corpus participants for the current study—as we wished to compare mouth activity in four cities in the United Kingdom, we examined 25 individuals from each of Glasgow, Belfast, Bristol, and London. Previous projects had already annotated certain parts of the corpus, and we used this as a starting point. In Bristol and London, only data from the conversation task had been annotated, whereas in Glasgow and Belfast, only narrative data had been annotated. Although as discussed in section 1.4.2, text-type can affect the rate of mouth activity in that narrative retellings tend to contain fewer mouthings, the narratives in the BSL Corpus are not retellings of existing stories as those from the Auslan Corpus: they are spontaneous/semi-spontaneous personal recounts. In fact, there is overlap between the personal narratives and spontaneous conversations in the BSL Corpus: many conversations naturally consist of some elements of personal narrative, and conversely, the personal narratives are often punctuated by interjections by the co-participant, as noted in Brown and Cormier (2017). Therefore, we concluded that this data selection would be appropriate for this study.

We considered variation based on the social factors taken into account during BSL Corpus creation. However, we excluded ethnicity because there were too few non-White individuals among the subset of participants chosen. The ethnic breakdown of our participants was 4 Asian, 4 Black, 1 Other, and 91 White. This broadly reflects the ethnic make-up of the UK population at the time of Corpus data collection in 2008–2010, based on the 2001 Census (the most recently available statistics for the United Kingdom at the time the project began): this was about 10% non-White, mostly Afro-Caribbean and south Asian origin. However, it has since become clear that the percentage of ethnic minorities in the deaf community may have been higher than this at the time of filming in 2008–2010. We highlight this issue to provide transparency in the description of our data collection. In any case, one would need more non-White participants in our subsample of participants to draw statistically sound conclusions around ethnicity. Also, we were unable to analyze social class as this cannot be dissociated from participants’ age because of the differing opportunities available to deaf people during the 20th century as a result of changes in discrimination legislation and deaf education policy (Schembri et al., 2013). Details of the distribution of participants according to the social factors under consideration are shown in Table 2.

**Table 2.** Distribution of Participants by City, Gender, Age and Language Background.

City	Gender		Age				Language background		Total
	Female	Male	16–35	36–50	51–64	65–88	Deaf relatives	Hearing relatives	
Belfast	13	12	7	6	9	3	6	19	25
Bristol	13	12	4	7	9	5	17	8	25
Glasgow	13	12	8	7	4	6	6	19	25
London	12	13	6	8	7	4	13	12	25
Total	51	49	25	28	29	18	42	58	100

### 3.2 Coding scheme

We examined the first (roughly) 100 sign tokens produced on the dominant hand of each of the 100 participants, to determine whether each was a verb. We tagged the mouth activity for those identified as verbs and recorded whether they were a plain or a non-plain verb. Similarly, we tagged any tokens made using the participant’s non-dominant hand. This approach means that it is not the case that the same number of verbs was identified for each participant, because they varied in the number of verbs produced during their first 100 tokens.

As this study focused on lexicalized plain and indicating verbs, we excluded tokens of constructed action, gesture, depicting signs, points, and fingerspelled items (glossed with a prefix of CA:, G:, DS:, PT: and FS: respectively as per Cormier et al., 2017). False starts, plus tokens containing / or ?, indicating uncertainty about the appropriate annotation, were also excluded.

#### 3.2.1 Identification of verbs

As this project was to investigate the rate of mouthing on verb signs, it was necessary to identify all the tokens of verbs in our subset of the corpus. This had already been done for the London and Bristol data for a different project (Fenlon et al., 2018). However, this was not the case for Glasgow and Belfast, so we undertook this task as part of the current study.

As discussed in Nadolske and Rosenstock (2007), determining the grammatical class of a sign in any sign language is not straightforward. This is because many signs can take more than one grammatical role, and this is not necessarily reflected in the morphology of the sign. Furthermore, the role of a particular token cannot be directly inferred based on its ID gloss (Johnston, 2012). In addition, the sequence of the signs in an utterance is not a reliable indicator because sign languages generally have a relatively flexible constituent order (Johnston et al., 2015). Therefore, we adopted a statistical approach, using the frequency with which a particular ID gloss had been identified as a verb in the parts of the corpus that were already tagged for grammatical category (as part of annotation for the study reported in Fenlon et al., 2018) as a starting point. We then made a judgment as to whether this categorization was appropriate for each token in context. In total 1,824 verb tokens were identified.

We tagged each token identified as a verb as to whether it was “plain” (body-anchored and not capable of spatial modification via path movement of the hands) or “non-plain.” So, for example, we identified support as non-plain because it is not body-anchored and it can be modified spatially toward its object. Similarly, give-up is non-plain, because it is not body-anchored. In contrast, think is a plain verb because it is body-anchored (at the forehead) and cannot be modified spatially.

#### 3.2.2 Categorization of mouth activity

We tagged each token identified as a verb in the parts of the corpus under consideration as having mouth activity categorized as “mouthing,” “mouth gesture” or “no/other mouth activity.” “Mouthing” was used if all or part of an English word was produced during that sign. We annotated mouth activity that did not appear to be derived from English as “mouth gesture,” although following Crasborn et al. (2008), we counted whole-face mouth gestures as “other” because they are not independent of other facial activity, as outlined in section 1.2. Where there was doubt as to the categorization of tokens of mouth activity, we consulted a deaf native BSL signer, to maximize our accuracy.

For mouthings, following Sutton-Spence and Day (2001) and Nadolske and Rosenstock (2007), we did not make a distinction as to whether a full English word or a reduced form of that word is produced. This was not a focus of the current project, and we felt that, in any case, it is not possible to determine the full extent of mouth activity by considering only the visible articulators. For alternative approaches, see Johnston et al. (2016) and Bank et al. (2011).

In terms of temporal alignment, previous studies have found that a given instance of mouth activity does not necessarily correspond exactly with a single manual sign. Some studies, such as Crasborn et al. (2008), have documented the exact onset and offset times of mouth activity but since the present project focused only on the mouth activity associated with verbs, we recorded the mouth activity corresponding to each such sign. We coded the mouth activity that occurred with at least 50% of the manual sign, following Johnston et al. (2016): in most cases, the context indicated that this corresponded to the manual sign in question but occasionally, it related to the preceding or following sign. Where mouth activity did not relate specifically to a verb sign but to the signs preceding or following them (which happened with 49 tokens), it was excluded from further analysis.

Finally, we excluded four tokens whose mouth activity was indecipherable because the participant’s mouth was not clearly visible on the video recording. This left 1,771 verb tokens to be analyzed for mouth activity. We conducted a consistency check, with an independent annotator (hearing, fluent BSL signer with sign linguistics training) examining a random 20% sample of annotations. We obtained a Cohen’s kappa chance-corrected agreement index (Cohen, 1960) of .818, indicating very good agreement between the annotations of each coder.

### 3.3 Statistical analysis

We employed generalized linear mixed effects modeling using R (R Core Team, 2020) with lme4 (Bates et al., 2015)^8^ to investigate the effect of each of the sociolinguistic factors under consideration on the rate of mouthing produced by participants. In each model, we included participant ID as a random intercept. This accounts for the fact that some individuals in the study may produce, for example, more mouthing than their social factor attributes would predict. These social factors are then only deemed significant if their effect is large enough to rise above the individual variation, meaning that this method reduces the chance of Type I errors.

## 4 Results

### 4.1 Rate of mouth activity, split by linguistic and social factors

#### 4.1.1 Overall

**Figure 1.**
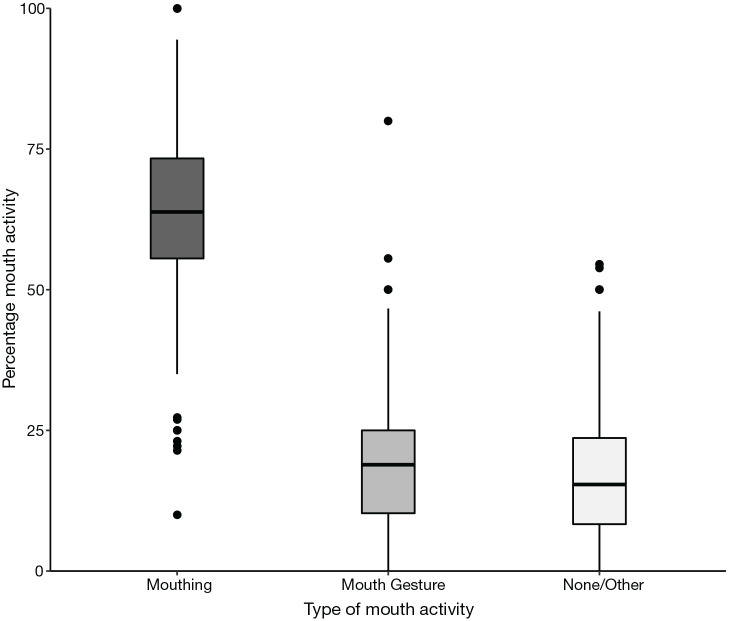
Percentage of mouth activity co-occurring with verbs, across all participants.

Figure 1 shows the overall rate of mouth activity across all participants studied. Mouthing ranged from 10% to 100% while mouth gestures ranged from 0% to 80%. “No/other mouth activity” ranged from 0% to 55%.

#### 4.1.2 Variation in mouth activity by participant

**Figure 2.**
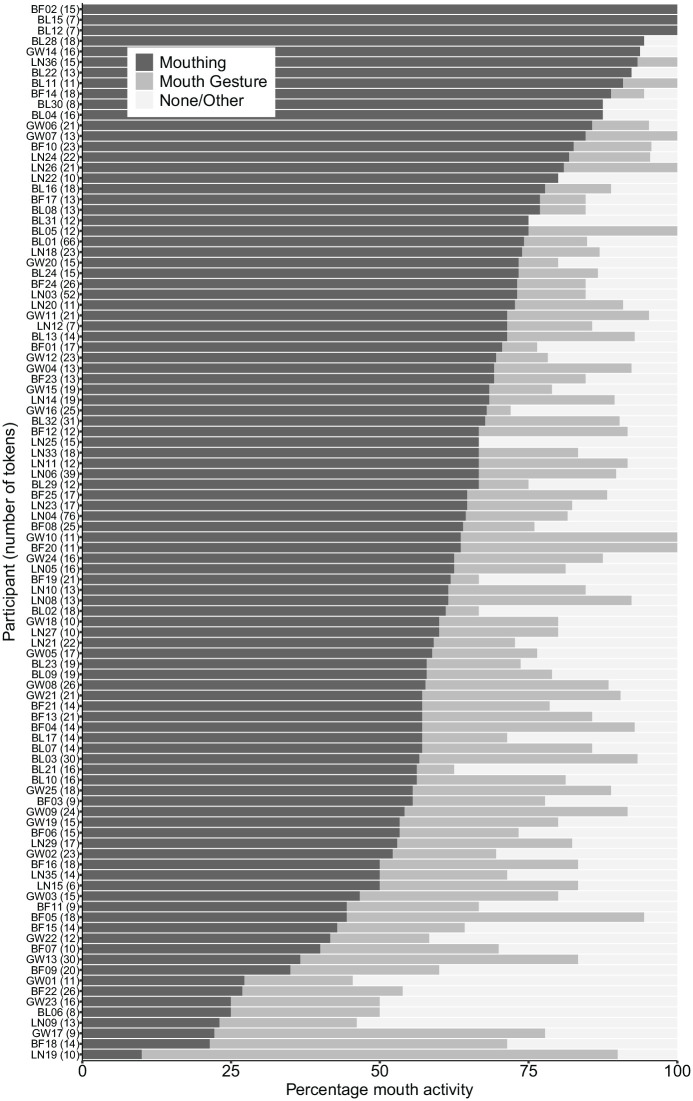
Percentage of mouth activity co-occurring with verbs, for each participant. *Note*. Sorted by percentage of mouthing. Number of tokens per participant is shown in brackets.

Figure 2 shows the variation in mouth activity between participants, ranked by increasing rate of mouthing. It shows that there is a great deal of variation, from a minimum of 10% mouthing to a maximum of 100%.

#### 4.1.3 Variation in mouth activity by sign

**Figure 3.**
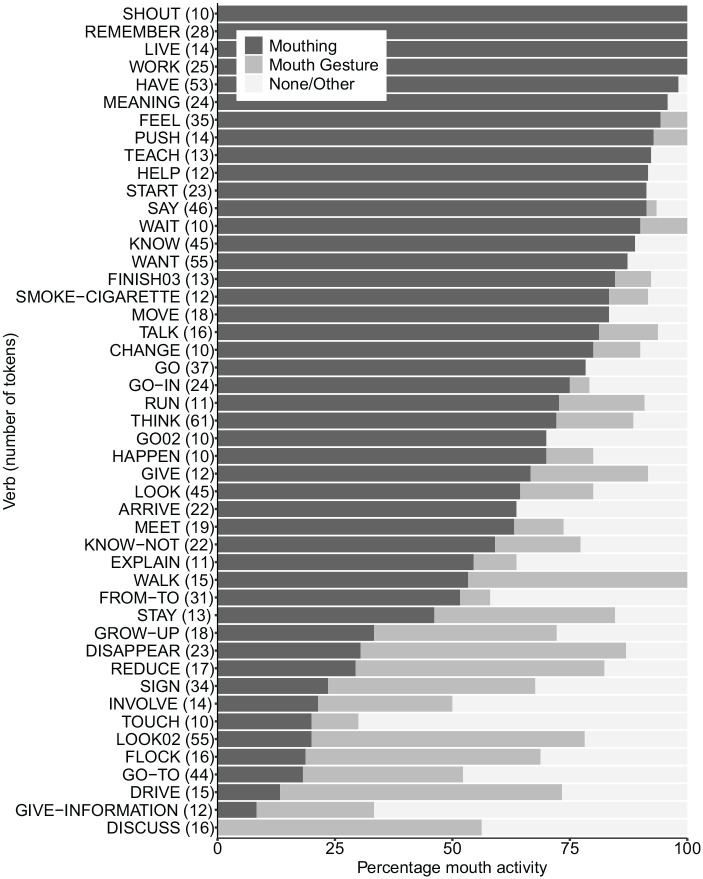
Breakdown of mouth activity associated with verbs occurring at least 10 times. *Note*. Sorted by percentage of mouthing. Number of tokens per sign shown in brackets.

Figure 3 provides a breakdown of the mouth activity on each verb in our sample that occurs at least 10 times.^9^ Where these were mouthings, the mouthings may be of all or part of any English translation equivalent of each ID gloss from BSL SignBank, and not necessarily mouthing of the ID gloss shown.^10^ It is clear that there is little homogeneity in the choice of mouthing, mouth gesture or “no/other mouth activity” for a given sign.

#### 4.1.4 Linguistic and social factors

**Figure 4.**
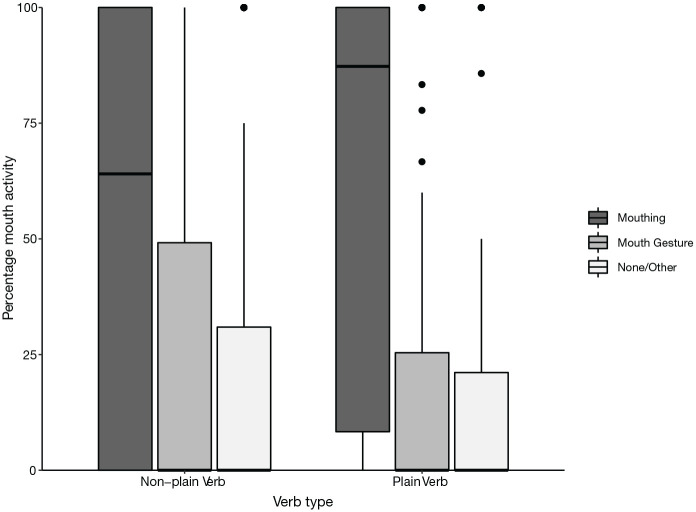
Percentage of mouth activity co-occurring with verbs, split by type of verb (plain vs. non-plain). *Note.* Difference between plain and non-plain verbs is significant.

**Figure 5.**
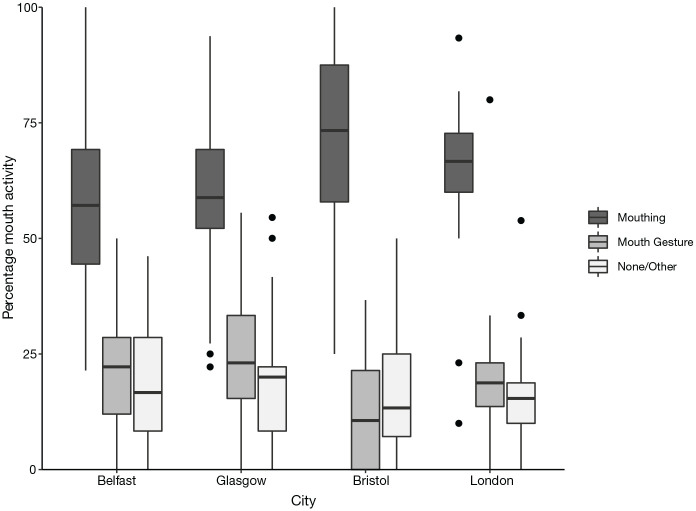
Percentage of mouth activity co-occurring with verbs, split by city. *Note.* Difference between cities is significant.

**Figure 6.**
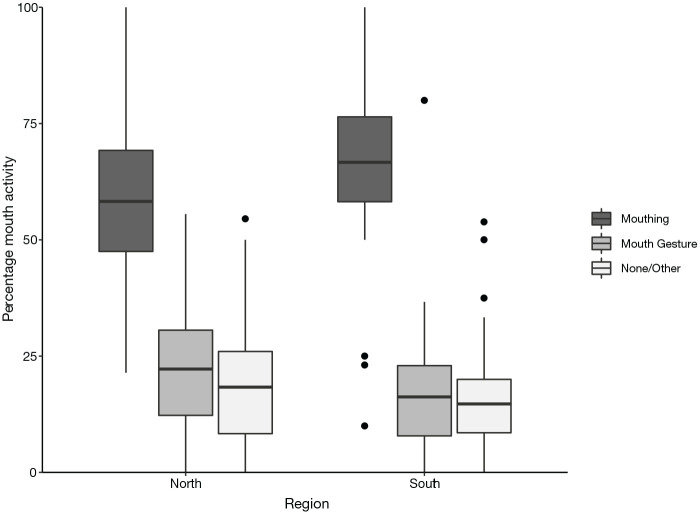
Percentage of mouth activity co-occurring with verbs, split by region (north/south). *Note*. Difference between regions is significant.

**Figure 7.**
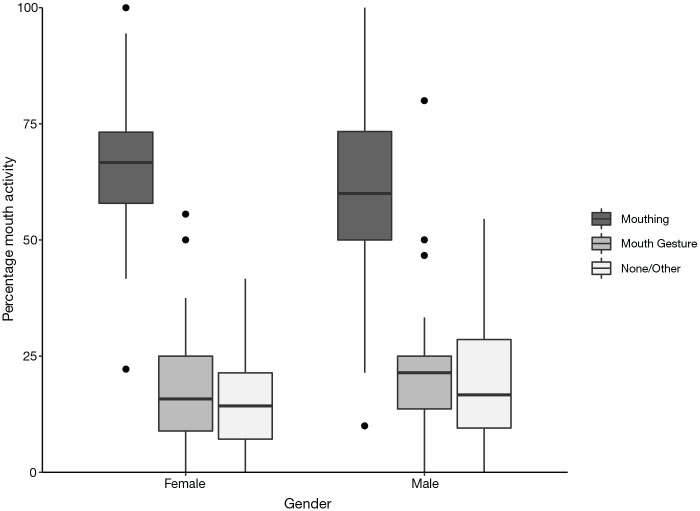
Percentage of mouth activity co-occurring with verbs, split by gender. *Note*. Difference between males and females is significant.

**Figure 8.**
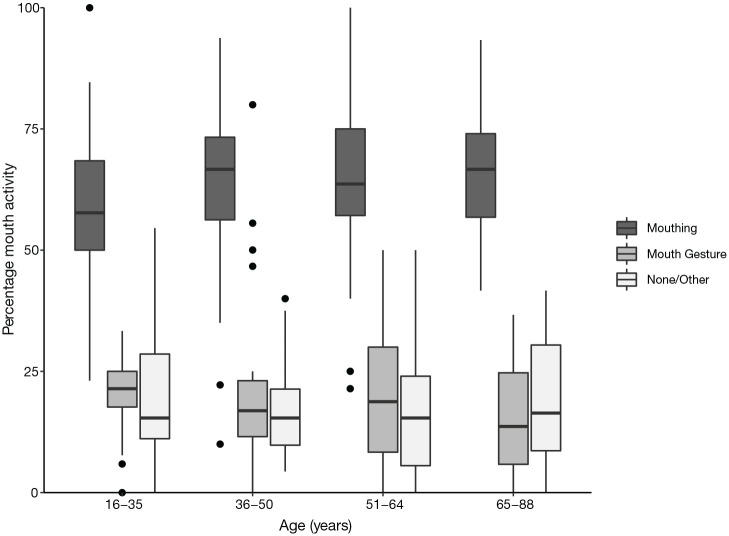
Percentage of mouth activity co-occurring with verbs, split by age category. *Note.* Difference between 16 to 35-year-olds and older participants is significant.

Figures 4 to 8 show the relative proportion of mouth activity on verbs, split by each of the linguistic and social factors under consideration in turn. Each graph shows the extent of mouth activity categorized as “mouthing,” “mouth gesture” and “no/other mouth activity.” For each factor, we report results of chi-square analyses using a threshold significance level of *p* = .05.

##### 4.1.4.1 Verb type

In terms of our linguistic factor, morphological complexity, we found a significant difference in mouth activity between plain and non-plain verbs as shown in Figure 4 (χ^2^ = 39.914, *df* = 1, *p* < .001, Cramer’s *V* = .140, which indicates a small to medium effect size). Each of mouthing, mouth gesture, and “no/other mouth activity” ranged from 0% to 100% for both plain and non-plain verbs.

##### 4.1.4.2 Region

Figure 5 shows mouth activity split into the four cities investigated. A chi-square analysis revealed that there is a significant difference in the rate of mouth activity between cities (χ^2^ = 17.108, *df* = 3, *p* = .0007, Cramer’s *V* = .098, which indicates a small effect size). In Belfast, mouthing ranged from 21% to 100% while mouth gestures ranged from 0% to 50%. “No/other mouth activity” ranged from 0% to 46%. In Glasgow, mouthing ranged from 22% to 94%, mouth gestures from 0% to 56%, and “no/other mouth activity” from 0% to 55%. In Bristol, mouthing ranged from 25% to 100%, mouth gestures from 0% to 37%, and “no/other mouth activity” from 0% to 50%. Finally, in London, mouthing ranged from 10% to 93%, mouth gestures from 0% to 80%, and “no/other mouth activity” from 0% to 54%.

Figure 6 shows the data combined for Belfast and Glasgow to represent the north of the United Kingdom, compared with combining London and Bristol to represent the south. The chi-square statistic remains significant: χ^2^ = 14.727, *df* = 1, *p* = .0001, Cramer’s *V* = .091, which still indicates a small effect size. In the north of the United Kingdom, mouthing ranged from 21% to 100%, mouth gestures from 0% to 56% and “no/other mouth activity” from 0% to 55%. In the south, mouthing ranged from 10% to 100%, mouth gestures from 0% to 80% and “no/other mouth activity” from 0% to 54%.

There is no significant difference between the rate of production of plain compared with non-plain verbs in the north versus the south of the United Kingdom (χ^2^ = 0.475, *df* = 1, *p* = .49 n.s., Cramer’s *V* = .016, which indicates a small effect size.) Therefore, this does not account for the difference in rate of mouthing between the north and the south.

##### 4.1.4.3 Gender

We found a significant difference in mouth activity between males and females in the study, as shown in Figure 7 (χ^2^ = 8.262, *df* = 1, *p* = .004, Cramer’s *V* = .068, which indicates a small effect size). In males, mouthing ranged from 10% to 100%, mouth gestures from 0% to 80%, and “no/other mouth activity” from 0% to 55%. In females, mouthing ranged from 22% to 100%, mouth gestures from 0% to 56%, and “no/other mouth activity” from 0% to 42%.

##### 4.1.4.4 Age

We investigated the mouth activity split by age group. Our hypothesis was that participants aged between 36 and 50 would produce more mouthings than those of other ages. However, this was not the case: there was no significant difference between people in this age group and all other participants (χ^2^ = 0.106, *df* = 1, *p* = .744 n.s., Cramer’s *V* = .008, which indicates a small effect size). A chi-square analysis of each age group considered separately revealed that there is a significant difference between the groups (χ^2^ = 8.779, *df* = 3, *p* = .032, Cramer’s *V* = .070, which indicates a small effect size). This is driven by participants aged between 16 and 35 years producing significantly less mouthing than those of other ages (χ^2^ = 7.657, *df* = 1, *p* = .006, Cramer’s *V* = .066, which indicates a small effect size). The rate of mouthing in all age groups is shown in Figure 8.

In participants aged between 16 and 35 years, mouthing ranged from 23% to 100%, mouth gestures from 0% to 33%, and “no/other mouth activity” from 0% to 55%. For those aged between 36 and 50 years, mouthing ranged from 10% to 94%, mouth gestures from 0% to 80%, and “no/other mouth activity” from 4% to 40%. People in the age range 51–64 years produced mouthings 21%–100% of the time, mouth gestures 0%–50% of the time and “no/other mouth activity” also 0%–50% of the time. Finally, those aged 65 years and older produced mouthings 42%–93% of the time, mouth gestures 0%–37% of the time, and “no/other mouth activity” 0%–42% of the time.

##### 4.1.4.5 Language background

There is no significant difference between the mouth activity produced by people with and without deaf relatives: χ^2^ = 0.786, *df* = 1, *p* = .375 n.s., Cramer’s *V* = .021, which indicates a small effect size. In those with deaf relatives, mouthing ranged from 23% to 100%, mouth gestures from 0% to 50%, and “no/other mouth activity” ranged from 0% to 55%. In those who reported no deaf relatives, mouthing ranged from 10% to 100%, mouth gestures ranged from 0% to 80%, and “no/other mouth activity” ranged from 0% to 46%.

### 4.2 Correlation between linguistic and social factors and mouthing

We created generalized linear mixed effects models with the binomial response family using trial-level data, first with our linguistic factor (verb type) as a fixed effect and then with the social factors that we found to be correlated with the rate of mouthing (region, gender, age group) as fixed effects. In each case, our dependent variable was the presence/absence of mouthing with each manual sign, with 0 = *no mouthing*, 1 = *mouthing*. We included participant as a random intercept and used a significance threshold of .05.

#### 4.2.1 Correlation between verb type and mouthing

Our linguistic factor, whether each verb was plain or non-plain, was correlated with the presence of mouthing. This is shown in Table 3: Plain verbs significantly favor mouthing whereas non-plain verbs significantly disfavor mouthing (*p* < .0001).

**Table 3.** Generalized Linear Mixed Effects Model of Effect of Verb Type on Rate of Mouthing.

Factor	Estimate	Std. Error	*z* value	Pr( >| z|)
(Intercept)	0.39328	0.08259	4.762	<.0001
Plain verb	0.70516	0.12451	5.664	<.0001

#### 4.2.2 Correlation between various social factors and mouthing

Our hypotheses were that we would observe more mouthings accompanying verbs in participants in the south of the United Kingdom compared with in the north and that women would produce more mouthings than men. Both these predictions were upheld. In terms of age, we predicted that people aged 36–50 years at the time of data collection would produce more mouthings than those in other age groups. This was not supported by our data: in fact, we found that those aged 16–35 years produced significantly fewer mouthings than the other participants. Therefore, we included membership of this age group (16–35 compared with older participants) in our model, along with region (north versus south of the United Kingdom) and gender (male vs. female). The model reveals that when these factors are considered together, only region has a significant effect on rate of mouthing (*p* = .0121); see Table 4.

**Table 4.** Generalized Linear Mixed Effects Model of Effect of Region, Gender and Age Group (Age 16–35 vs. Other Ages) on Rate of Mouthing.

Factor	Estimate	Std. Error	*z* value	Pr( >| z|)
(Intercept)	0.9473	0.1279	7.407	<.0001
Region: north	−0.3660	0.1459	−2.509	.0121
Gender: male	−0.2766	0.1476	−1.873	.0610
Age: 16–35	−0.2050	0.1688	−1.214	.2248

## 5 Discussion

Overall, we found considerable variation between participants in the amount of mouthing that they produced with verb signs, and similarly, a high degree of variation in the rate of mouthing with particular verb signs. In addition, we observed a strong association between verb type and rate of mouthing, with mouthing produced significantly more with plain than non-plain verbs. Of our social factors, we found that region, gender, and age were significant, with individuals in the south of the United Kingdom producing more mouthings with verbs than those in the north, women producing more mouthings with verbs than men, and people aged 16–35 years producing fewer mouthings than older participants. We now discuss how these results relate to our hypotheses and to previous studies.

### 5.1 Variation in mouth activity

There is a great deal of variation between participants in the proportions of mouthing that they produce. Figure 2 illustrates the degree of variability between individuals. Similar effects have been found in other studies: for example, Nadolske and Rosenstock (2007) found in their study of ASL storytelling that the rate of mouthing across only seven retellings ranged from below 30% to 60%.

A high degree of individual variation was also reported in Johnston et al. (2016). Although their data cover all grammatical categories and four sign languages (Auslan, BSL, NGT, and Swedish Sign Language), whereas the present study considers only verbs in BSL, a remarkably similar pattern and degree of variation is observed in comparison to the current study. This is despite the fact that the Auslan data in their study amounted to over 17,000 tokens, nearly 10 times larger than the current project (1,771). They also included fewer participants—38 rather than 100. The larger number of tokens per participant in the Auslan study means that the mouth activity distribution for each individual is less prone to sampling error. The fact that we studied mouthing in fewer tokens per participant but in more individuals, and still found a similar amount of individual variation as the Auslan study, strengthens this overall finding across the studies.

In terms of variation per sign, it is clear from Figure 3 that there is generally no standard choice of mouthing, mouth gesture, or “no/other mouth activity” per verb in BSL, as was observed by Bank et al. (2011) for NGT. This, and the high degree of variation in mouth activity per participant, clearly shows that mouthings on verbs in BSL are not compulsory. This leads us to the conclusion that participants are (subconsciously) choosing in real-time what mouth activity to combine with each manual sign and is consistent with the theory that where they choose mouthing, this constitutes code-blending between English and BSL.

### 5.2 Verb type

Looking at our linguistic factor, we found as we predicted that the rate of mouthing is inversely proportional to the morphological complexity of the accompanying verb sign. Verb type is a highly significant predictor, with a markedly larger amount of mouthing observed on plain verbs (75%) compared with non-plain verbs (59%). This supports general findings from previous studies covering a range of sign languages, as discussed in section 1.4.1.

However, looking at studies that quote specific figures for mouth activity on plain and non-plain verbs, we see some differences. Auslan and BSL are very closely related languages: in fact Johnston (2001) argues that they are dialects of the same language. Therefore, we may expect similar results in our study compared with Johnston et al. (2016), yet their findings are very different, as shown in Tables 5 and 6.

**Table 5.** Comparison of Percentage Mouth Activity on Plain Verbs between Current Study and Johnston et al. (2016).

Plain Verbs	Mouthings (%)	Mouth gestures (%)	“None/other” (%)	Number of tokens
Johnston et al. (2016)	34	44	22	329
Current study	75	14	11	465

**Table 6.** Comparison of Percentage Mouth Activity on Non-Plain Verbs between Current Study and Johnston et al. (2016).

Non-plain verbs	Mouthings (%)	Mouth gestures (%)	“None/other” (%)	Number of tokens
Johnston et al. (2016)	36	42	22	1,004
Current study	59	21	20	1,316

For both plain and non-plain verbs, our study found a far higher proportion of mouthings and a far lower proportion of mouth gestures. This may be explained by the fact that the overall Auslan Corpus, upon which their study was based, contains a large amount of narrative-retelling rather than spontaneous personal narratives. As discussed in section 1.4.2, narrative retellings are likely to contain more constructed action (and therefore mouth gesture) than conversation. Since Johnston et al. do not report what proportion of the subset of their corpus is narrative retelling, we cannot be sure of the size of this effect.

### 5.3 Region

Our hypothesis relating to region is supported by our findings: the rate of mouthing in the two cities in the north of the United Kingdom (59%) is significantly lower than the two cities in the south (68%) (although the effect size is small), even though there was no significant difference in the amount of plain or non-plain verbs produced in the north compared with the south. The difference is driven by the fact that participants from Bristol produced fewer mouth gestures than those in the rest of the country: mouth gestures made up 14% of Bristol participants’ mouth activity compared with 21% for Belfast. However, because the project compared conversation data in the south with personal narrative data in the north, further research is required to establish whether it is the regional difference or the text-type difference that is correlated with the observed effect. The results of Rentelis (2011) are supported by this work: Rentelis looked at conversation data from both the north and south of the United Kingdom, but our study considered more cities and more than three times as many tokens.

### 5.4 Gender

A significant effect of gender was observed, supporting our hypothesis. The rate of mouthing among males (60%) was significantly lower than that among females (67%), though as for region, the effect size is small. This confirms the effect that Rentelis (2011) found with his smaller data sample. As discussed by Rentelis and by Ladd (2003), the greater use of mouthings by women may be because the use of mouthings with BSL is considered to have higher prestige, and women are known to adopt prestige language forms ahead of men. Ladd identifies working-class and middle-class groups within the British deaf community and suggests that middle-class deaf people aspire to speaking rather than signing when in the company of hearing people. Since mouthings in BSL are influences of spoken English, signing with mouthing could be said to be a prestige variety of BSL. The fact that Labov (2001) claims that women adopt prestige forms ahead of men might therefore explain why women use mouthings more than men.

### 5.5 Age

We had expected to find that signers aged 36–50 years would use significantly more mouthings than the other participants, because of the greater use of spoken English during their education. But this was not the case: the rate of mouthing among these participants (64%) was almost identical to the rate in all the other age groups (63%). Instead, we found that people aged 16–35 years produced significantly less mouthing (58%) than those of other ages (65%), once again, with a small effect size. According to the apparent time hypothesis (Bailey et al., 1991), the difference between age groups suggests that the rate of mouthing in BSL may have begun to reduce, perhaps as a result of the changes in deaf education policy over the past century.

### 5.6 Language background

We hypothesized that whether a participant had deaf family members would not affect their rate of mouthing with verbs, and this was supported by the data. The rate of mouthings among people with at least one deaf relative (65%) is not significantly different from those with only hearing family members (63%).

### 5.7 Overall rate of mouth activity with verbs

The overall rate of mouth activity in this study was 64% mouthing, 19% mouth gesture, and 17% no/other mouth activity. Although this study covered only mouth activity with verbs, its findings are broadly consistent with past research covering a range of sign languages on rate of mouthing: in general, we found more mouthing than mouth gestures or no/other mouth activity (cf. Table 1).

Considering the BSL data specifically, the most striking observation is the high degree of similarity between the current study and that of Sutton-Spence and Day (2001). This is even though the earlier work considers all grammatical categories of sign rather than just verbs, and despite the fact that it covers many different text-types. It may be that individual variation has been evened out because both studies used a relatively large number of participants (39 for Sutton-Spence and Day, 100 for the current study).

By contrast, the BSL participants from Crasborn et al. (2008) produced mouthings at about half the rate that we observed. This may be due to the lower rate of mouthing generally associated with narrative retelling since Crasborn et al. used a storytelling task, whereas our study involved free conversation and personal narratives. Narrative retellings often include a relatively large amount of constructed action (i.e., enactment demonstrating actions of a referent: see Cormier et al., 2015). Mouth gestures are a key part of overt instances of constructed action (Johnston et al., 2016), and in these cases, fewer mouthings can co-occur. In addition, Crasborn et al.’s participants were experienced storytellers (cf. Earis & Cormier, 2013) so they may have used more constructed action and/or mouth gestures in their dramatic portrayal, whereas the BSL Corpus participants were not recruited for storytelling experience. In addition, the BSL Corpus data were collected in a way that encouraged production of “vernacular BSL” (Schembri et al., 2013), which involves some influence from English. Crasborn et al.’s participants may have used a more “self-conscious” style of BSL, including “hypercorrection” (Schembri et al., 2013) to reduce English influence, perhaps because they knew that their production was to be archived as examples of high-quality BSL storytelling.

### 5.8 Theoretical implications

We found a high degree of variation between participants in the rate of mouthing production with verbs, and a lack of consistency in the choice of mouth activity with a given verb. We observed only weak correlations, with small effect sizes, between rate of mouthing and participants’ region, gender, and age and no correlation between rate of mouthing and participants’ language background. All this is consistent with the position that mouthings are not an inherent compulsory part of the phonological or lexical specification of a sign: instead, they are better considered as independent units originating from the ambient spoken language that can be combined simultaneously with the manual sign.

In previous sign language research, this has been treated as a process of “code-blending,” considered to be typologically unique to sign languages. We argue that instead, code-blending is best considered as a type of translanguaging that occurs with sign languages and their ambient spoken language(s). In this sense, code-blending is not that different from what happens when speakers mix various elements of their linguistic repertoires, particularly when they do so multimodally and/or simultaneously. This is consistent with other recent works on translanguaging in deaf signers which mention mouthing as one of the elements that is often included when signers mix multimodal linguistic repertoires (De Meulder et al., 2019; Hodge & Goswell, 2021; Kusters et al., 2017). We agree with Bank et al. (2011) that code-blending is typologically different from code-switching in spoken languages. This is because code-switching (whether considered as a type of translanguaging or not) is an inherently sequential process—switching linearly between two linguistic elements. The multiple articulators available in signed language (the mouth as well as the hands) mean that two languages can be produced at the same time. This is not possible in the same way for prototypical code-switching in spoken languages because of the sequential nature of speech: The nearest equivalent might be phenomena that are not considered code-switching at all such as the influence across languages of suprasegmental features such as tone, pitch accent, or stress that can occur when words are borrowed from one language to another (e.g., Kang, 2010). Kang observes that when words are borrowed, a range of strategies can be employed. For borrowing into tone or pitch accent or stress accent languages, sometimes it is not possible to preserve the suprasegmental features of the borrowed language. Instead, characteristics of the matrix language can be applied to the borrowed word so that features of both languages are produced at the same time. For example, when “Chrístmas” is borrowed from English into Japanese (a pitch accent language), *kurisúmasu* is produced using the default accent of Japanese rather than retaining the stress found in English (Kang, 2010, p. 2302). A multimodal notion of translanguaging encompasses all of these patterns regardless of modality or degree of conventionality (including intonation, writing, and gesture) (Kusters et al., 2017; Wei, 2018b), and also regardless of sequential/simultaneous structure.

### 5.9 Future research

This study focussed primarily on mouthing with BSL verb signs: future studies could consider the distribution of mouthing, mouth gestures, and no/other mouth activity in other grammatical categories of sign in BSL. We compared mouth activity used in conversation data for the south of the United Kingdom with narrative data from the north, so further research using a single text-type from the BSL Corpus would establish whether the regional difference in mouthing we observed is due to this text-type difference or due to a genuine geographic variation.

Future studies of mouthing in BSL could investigate which English words (or which parts of words) are mouthed with particular signs, or the extent to which the same temporal reductions in a given mouthing are used across sign tokens and across signers.

The effect of sign frequency on mouth activity could also be explored. In addition, future studies could consider the temporal alignment of mouthings with the manual component of the sign. A high degree of variation in these areas would provide stronger evidence that mouthing is an example of code-blending between the sign language and the ambient spoken language, because it would suggest that the signer has more freedom over which mouthing (if any), and how much of it, to produce with a manual sign, as discussed in Bank et al. (2011).

Other social factors could also be considered in future work on mouthing in BSL. As noted in section 3.1, ethnicity and social class were not included in the current study: further investigation of the role of mouthing with these social factors would be useful. Studying social class and mouthing might help explain some of our findings regarding gender differences as noted in section 5.4. With ethnicity, very little has been reported on use of mouthing and ethnicity in sign languages, though McCaskill et al. (2011) found in their study of Black ASL that older Deaf Black signers of ASL use mouthing less than White Deaf signers (and also younger Deaf Black signers). It would be useful to study the role of ethnicity in the use of mouthing (and any similar interactions with other social factors) in BSL as well.

In addition to mouthing, the use of fingerspelling is another influence from the surrounding spoken language upon sign language. Mouthings and fingerspellings are inter-linked, in that mouthings can be used to disambiguate fingerspelling sequences (Brown & Cormier, 2017). It was not possible to work directly with the survey of fingerspelling carried out by Brown and Cormier because the present study concentrated on lexical verbs and so excluded non-lexicalized fingerspelling. In terms of future research, one could investigate, for example, the percentage of mouthings that occur with fingerspellings, and conversely, the percentage of fingerspellings that are mouthed.

Mouthing and fingerspelling are two major types of influence from the ambient spoken language on sign languages. There are others, such as influence from spoken language grammatical structures (e.g., constituent order). It would be useful in future work to consider all of these phenomena under the lens of translanguaging from a theoretical perspective and how this compares with multimodal translanguaging practices in hearing non-signers.

### 6 Conclusion

Our study has examined extracts from the BSL Corpus to determine the effect of various sociolinguistic factors on the rate of mouthing accompanying verb signs in BSL. In terms of linguistic factors, our hypothesis was that mouthing occurs more frequently with plain verbs than with indicating or depicting verbs, and this was strongly supported. We suggest that this is because a translation equivalent of a given non-plain verb is often a phrase in English (rather than a single word), which cannot be incorporated in a single mouthing. Therefore, mouth gestures are more likely to accompany such morphologically complex verbs. Although this has previously been observed in BSL using a range of smaller datasets, and in Auslan using a corpus consisting mainly of narrative retellings, this is the first time that such research has been undertaken using a corpus of spontaneous sign language conversation and personal narrative data.

The social factors that we considered were region, gender, age, and language background. We found no effect of language background. We found a significant gender difference: women produce more mouthings with verbs than men. This may be because use of mouthings is associated with a higher prestige form of BSL, and the fact that women tend to adopt prestige forms ahead of men. In terms of region, we used a larger sample size, using more regions than was possible in a previous study using the BSL Corpus, Rentelis (2011), and our findings back-up and extend this research. We conclude that mouthings are used significantly more by signers in the south of the United Kingdom (London and Bristol) compared with those in the north (Glasgow and Belfast), although we note that further research is needed to establish whether this is a genuine regional difference. We find that the youngest signers in our cohort, aged 16–35 years, produced significantly less mouthing than older participants, suggesting that the rate of mouthing in BSL may be decreasing over time.

We also observed a high degree of variation in the rate of mouthing with verbs, both between participants and with particular signs. This supports the position that mouthings are not part of the phonological or lexical specification of signs and suggests that they should instead be considered as elements from the ambient spoken language, selected for use with manual signs in a process of code-blending. In addition, we suggest that code-blending should be considered as a type of translanguaging. This would account for production of two languages simultaneously and/or sequentially within a single utterance, regardless of language modality.

## Supplemental Material

sj-csv-1-las-10.1177_00238309221107002 – Supplemental material for Sociolinguistic Variation in Mouthings in British Sign Language: A Corpus-Based Study Click here for additional data file. Supplemental material, sj-csv-1-las-10.1177_00238309221107002 for Sociolinguistic Variation in Mouthings in British Sign Language: A Corpus-Based Study by Heidi Proctor and Kearsy Cormier in Language and Speech

sj-csv-2-las-10.1177_00238309221107002 – Supplemental material for Sociolinguistic Variation in Mouthings in British Sign Language: A Corpus-Based Study Click here for additional data file. Supplemental material, sj-csv-2-las-10.1177_00238309221107002 for Sociolinguistic Variation in Mouthings in British Sign Language: A Corpus-Based Study by Heidi Proctor and Kearsy Cormier in Language and Speech

sj-html-1-las-10.1177_00238309221107002 – Supplemental material for Sociolinguistic Variation in Mouthings in British Sign Language: A Corpus-Based Study Click here for additional data file. Supplemental material, sj-html-1-las-10.1177_00238309221107002 for Sociolinguistic Variation in Mouthings in British Sign Language: A Corpus-Based Study by Heidi Proctor and Kearsy Cormier in Language and Speech

sj-rmd-1-las-10.1177_00238309221107002 – Supplemental material for Sociolinguistic Variation in Mouthings in British Sign Language: A Corpus-Based Study Click here for additional data file. Supplemental material, sj-rmd-1-las-10.1177_00238309221107002 for Sociolinguistic Variation in Mouthings in British Sign Language: A Corpus-Based Study by Heidi Proctor and Kearsy Cormier in Language and Speech

## Notes

1 Mouthings are not spoken English: They are silent mouthings of some or all of an English word produced while signing.

2 Fingerspelling is the use of specific signs to denote each letter of the alphabet to spell out English words.

3 For a definition of plain verbs, see section 1.4.1.

4 Liddell’s (2003) class of indicating verbs includes what Padden (1983) refers to as agreement or agreeing verbs, and also some of what Padden considers spatial verbs (i.e., all spatial verbs aside from depicting/classifier verbs).

5 There appears to be a discrepancy in their publication between this figure and the values in the corresponding table (p. 80), which indicate that 60% of narrative signs are accompanied by mouthing.

6 Sign-supported English (SSE) combines signs with spoken English, using the grammar of spoken English rather than of BSL.

7 Some studies explicitly excluded mouth activity that occurred without a manual sign, where others included this. Still others were not explicit on this point.

8 Please contact the first author for a copy of this R code plus anonymized mouth activity data.

9 We excluded signs that occurred fewer than 10 times to reduce the bias caused by a low frequency of occurrence.

10 As noted in section 3.2.1, ID glosses do not reflect grammatical function. For example, the ID gloss from-to is used for the sign that can mean “attend,” “attendance,” “go,” “come,” “going,” “from,” “to,” and so forth, regardless of whether the token in question is functioning as a verb or noun or preposition. The 31 tokens of from-to shown in Figure 3 were all functioning as verbs.

## References

[bibr1-00238309221107002] Ajello R. Mazzoni L. Nicolai F. (2001). Linguistic gestures: mouthings in Italian Sign Language (LIS). In Braem P. Boyes Sutton-Spence R. (Eds.), The hands are the head of the mouth: The mouth as articulator in sign languages (pp. 231–246). Signum Press.

[bibr2-00238309221107002] Auer P. (under review). “Translanguaging” or “doing languages”? Multilingual practices and the notion of “codes.” In MacSwan J. (Ed.), Language(s): Multilingualism and Its Consequences.

[bibr3-00238309221107002] Bailey G. Wikle T. Tillery J. Sand L. (1991). The apparent time construct. Language Variation and Change, 3, 241–264. 10.1017/S0954394500000569

[bibr4-00238309221107002] Bank R. (2015). The ubiquity of mouthings in NGT: A corpus study [Doctoral dissertation, Radboud University Nijmegen]. http://hdl.handle.net/2066/133714

[bibr5-00238309221107002] Bank R. Crasborn O. van Hout R. (2011). Variation in mouth actions with manual signs in Sign Language of the Netherlands (NGT). Sign Language & Linguistics, 14(2), 248–270. 10.1075/sll.14.2.02ban

[bibr6-00238309221107002] Bank R. Crasborn O. van Hout R. (2015). Alignment of two languages: The spreading of mouthings in Sign Language of the Netherlands. International Journal of Bilingualism, 19(1), 40–55. 10.1177/1367006913484991

[bibr7-00238309221107002] Bates D. Mächler M. Bolker B. Walker S. (2015). Fitting linear mixed-effects models using lme4. Journal of Statistical Software, 67(1), 1–48. 10.18637/jss.v067.i01

[bibr8-00238309221107002] Boyes Braem P . (2001). Functions of the mouthings in the signing of deaf early and late learners of Swiss German Sign Language (DSGS). In Boyes Braem P. Sutton-Spence R. (Eds.), The hands are the head of the mouth: The mouth as articulator in sign languages (pp. 99–131). Signum Press.

[bibr9-00238309221107002] Boyes Braem P. Sutton-Spence R . (2001). The hands are the head of the mouth: The mouth as articulator in sign languages. Signum Press.

[bibr10-00238309221107002] Brown M. Cormier K. (2017). Sociolinguistic variation in the nativisation of BSL fingerspelling. Open Linguistics, 3(1), 115–144. 10.1515/opli-2017-0007

[bibr11-00238309221107002] Chan B. (2021). Translanguaging or code-switching?: Reassessing mixing of English in Hong Kong Cantonese. Chinese Language and Discourse. 10.1075/cld.20003.cha

[bibr12-00238309221107002] Cohen J. (1960). A coefficient of agreement for nominal scales. Educational and Psychological Measurement, 20(1), 37–46.

[bibr13-00238309221107002] Cormier K. Fenlon J. Gulamani S. Smith S. (2017, March). BSL Corpus annotation conventions: Version 3.0. https://bslcorpusproject.org/wp-content/uploads/BSLCorpus_AnnotationConventions_v3.0_-March2017.pdf

[bibr14-00238309221107002] Cormier K. Quinto-Pozos D. Sevcikova Z. Schembri A. (2012). Lexicalisation and de-lexicalisation processes in sign languages: Comparing depicting constructions and viewpoint gestures. Language and Communication, 32(4), 329–348. 10.1016/j.langcom.2012.09.004 23805017 PMC3688355

[bibr15-00238309221107002] Cormier K. Smith S. Sevcikova-Sehyr Z. (2015). Rethinking constructed action. Sign Language & Linguistics, 18(2), 167–204. 10.1075/sll.18.2.01cor

[bibr16-00238309221107002] Crasborn O. van der Kooij E. Waters D. Woll B. Mesch J. (2008). Frequency distribution and spreading behavior of different types of mouth actions in three sign languages. Sign Language & Linguistics, 11(1), 45–67. 10.1075/sl&l.11.1.04cra

[bibr17-00238309221107002] Day L. (1995). Sign language acquisition of deaf adults in deaf and hearing Families [Unpublished Video Manuscript]. University of Bristol.

[bibr18-00238309221107002] De Meulder M. Kusters A. Moriarty E. Murray J. J . (2019). Describe, don’t prescribe. The practice and politics of translanguaging in the context of deaf signers. Journal of Multilingual and Multicultural Development, 40(10), 892–906. 10.1080/01434632.2019.1592181

[bibr19-00238309221107002] de Vos C. Zeshan U . (2012). Introduction: Demographic, sociocultural, and linguistic variation across rural signing communities. In de Vos C. Zeshan U. (Eds.), Sign languages in village communities: Anthropological and linguistic insights (pp. 2–24). De Gruyter Mouton. 10.1515/9781614511496.2

[bibr20-00238309221107002] Earis H. Cormier K. (2013). Point of view in British Sign Language and spoken English narrative discourse: The example of “The Tortoise and the Hare.” Language and Cognition, 5(4), 313–343. 10.1515/langcog-2013-0021

[bibr21-00238309221107002] Ebbinghaus H. Hessmann J. (2001). Sign Language as multidimensional communication: Why manual signs, mouthings and mouth gestures are three different things. In Boyes Braem P. Sutton-Spence R. (Eds.), The hands are the head of the mouth: The mouth as articulator in sign languages (pp. 133–151). Signum Press.

[bibr22-00238309221107002] Emmorey K. Borinstein H. B. Thompson R. (2005). Bimodal bilingualism : Code-blending between spoken English and American Sign Language. In Cohen J McAlister K. T. Rolstad K. MacSwan J. (Eds.), ISB4: Proceedings of the 4th International Symposium on Bilingualism (pp. 663–673). Cascadilla Press.

[bibr23-00238309221107002] Fenlon J. Schembri A. Cormier K. (2018). Modification of indicating verbs in British Sign Language: A corpus-based study. Language, 94(1), 84–118. 10.1353/lan.2018.0002

[bibr24-00238309221107002] Fontana S. (2008). Mouth actions as gesture in sign language. Gesture, 8(1), 104–123. 10.1075/gest.8.1.08fon

[bibr25-00238309221107002] García O. (2009). Education, multilingualism and translanguaging in the 21st century. In Skutnabb-Kangas T. Phillipson R. Mohanty A. K. Panda M. (Eds.), Social Justice through Multilingual Education (pp. 140–158). Multilingual Matters. 10.21832/9781847691910-011

[bibr26-00238309221107002] Giustolisi B. Mereghetti E. Cecchetto C. (2017). Phonological blending or code mixing? Why mouthing is not a core component of sign language grammar. Natural Language and Linguistic Theory, 35(2), 347–365. 10.1007/s11049-016-9353-9

[bibr27-00238309221107002] Hodge G. Goswell D. (2021). Deaf signing diversity and signed language translations. Applied Linguistics Review. 10.1515/applirev-2020-0034

[bibr28-00238309221107002] Hohenberger A. Happ D. (2001). The linguistic primacy of signs and mouth gestures over mouthings: Evidence from language production in German Sign Language (DGS). In Boyes Braem P. Sutton-Spence R. (Eds.), The hands are the head of the mouth: The mouth as articulator in sign languages (pp. 153–189). Signum Press.

[bibr29-00238309221107002] Johnston T. (2001). The lexical database of Auslan (Australian Sign Language). Sign Language & Linguistics, 4(1), 145–169. 10.1075/sll.4.12.11joh

[bibr30-00238309221107002] Johnston T. (2012). Lexical frequency in sign languages. Journal of Deaf Studies and Deaf Education, 17(2), 163–193. 10.1093/deafed/enr036 21841168

[bibr31-00238309221107002] Johnston T. Cresdee D. Schembri A. Woll B. (2015). FINISH variation and grammaticalization in a signed language: How far down this well-trodden pathway is Auslan (Australian Sign Language)? Language Variation and Change, 27, 117–155. 10.1017/S0954394514000209

[bibr32-00238309221107002] Johnston T. Schembri A. (2007). Australian Sign Language (Auslan): An introduction to sign language linguistics. Cambridge University Press. 10.1017/CBO9780511607479

[bibr33-00238309221107002] Johnston T. van Roekel J. Schembri A. (2016). On the conventionalization of mouth actions in Australian Sign Language. Language and Speech, 59(1), 3–42. 10.1177/0023830915569334 27089804

[bibr34-00238309221107002] Kang Y. (2010). Tutorial overview: Suprasegmental adaptation in loanwords. Lingua, 120(9), 2295–2310. 10.1016/j.lingua.2010.02.015

[bibr35-00238309221107002] Kusters A. Spotti M. Swanwick R. Tapio E. (2017). Beyond languages, beyond modalities: Transforming the study of semiotic repertoires. International Journal of Multilingualism, 14(3), 219–232. 10.1080/14790718.2017.1321651

[bibr36-00238309221107002] Labov W. (2001). Principles of linguistic change, Vol. 2: Social factors. Blackwell.

[bibr37-00238309221107002] Ladd P. (2003). Understanding deaf culture: In search of Deafhood. Multilingual Matters.

[bibr38-00238309221107002] Lewin D. Schembri A. C. (2011). Mouth gestures in British Sign Language: A case study of tongue protrusion in BSL narratives. Sign Language & Linguistics, 14(1), 94–114. 10.1075/sll.14.1.06lew

[bibr39-00238309221107002] Liddell S. K. (2003). Grammar, gesture, and meaning in American Sign Language. Cambridge University Press.

[bibr40-00238309221107002] Lucas C. (Ed.). (2001). The sociolinguistics of sign languages. Cambridge University Press. 10.1017/CBO9780511612824

[bibr41-00238309221107002] MacSwan J. (2000). The architecture of the bilingual language faculty: Evidence from intrasentential code switching. Bilingualism: Language and Cognition, 3(1), 37–54. 10.1017/s1366728900000122

[bibr42-00238309221107002] MacSwan J. (Ed.). (2014). Grammatical theory and bilingual codeswitching. MIT Press. 10.7551/mitpress/9780262027892.001.0001

[bibr43-00238309221107002] Mayberry R. I. (2007). When timing is everything: Age of first-language acquisition effects on second-language learning. Applied Psycholinguistics, 28(3), 537–549.

[bibr44-00238309221107002] McCaskill C. Lucas C. Bayley R. Hill J. (2011). The hidden treasure of Black ASL: Its history and structure. Gallaudet University Press.

[bibr45-00238309221107002] Meir I. (2012). Word classes and word formation. In Pfau R. Steinbach M. Woll B. (Eds.), Sign Language—An international handbook (pp. 77–111). De Gruyter Mouton.

[bibr46-00238309221107002] Mitchell R. E. Karchmer M. A. (2004). Chasing the mythical ten percent: Parental hearing status of deaf and hard of hearing students in the United States. Sign Language Studies, 4(2), 138–163. 10.1353/sls.2004.0005

[bibr47-00238309221107002] Mohr S. (2012). The visual-gestural modality and beyond: Mouthings as a language contact phenomenon in Irish Sign Language. Sign Language & Linguistics, 15(2), 185–211. 10.1075/sll.15.2.01moh

[bibr48-00238309221107002] Myers-Scotton C. (1993). Duelling languages: Grammatical structure in codeswitching. Clarendon Press.

[bibr49-00238309221107002] Nadolske M. A. Rosenstock R. (2007). Occurrence of mouthings in American Sign Language: A preliminary study. In Perniss P. M. Pfau R. Steinbach M. (Eds.), Visible variation (pp. 35–61). De Gruyter Mouton.

[bibr50-00238309221107002] Padden C. (1983). Interaction of morphology and syntax in American Sign Language. University of California, San Diego.

[bibr51-00238309221107002] Poplack S. (1980). Sometimes I’ll start a sentence in Spanish Y TERMINO EN ESPAÑOL: Toward a typology of code-switching. Linguistics, 18(7–8), 581–618. 10.1515/ling.1980.18.7-8.581

[bibr52-00238309221107002] Poplack S. (2018). Borrowing: Loanwords in the speech community and in the grammar. Oxford University Press. 10.1093/oso/9780190256388.001.0001

[bibr53-00238309221107002] R Core Team. (2020). R: A language and environment for statistical computing. https://www.r-project.org/

[bibr54-00238309221107002] Rentelis R. (2011). British Sign Language mouthings from the north and south of the UK [Unpublished master’s thesis]. University of Bristol.

[bibr55-00238309221107002] Sandler W. (2012). Dedicated gestures and the emergence of sign language. Gesture, 12(3), 1–41.

[bibr56-00238309221107002] Schembri A. Fenlon J. Rentelis R. Cormier K. (2014). British Sign Language corpus project: A corpus of digital video data and annotations of British Sign Language 2008–2014 (2nd ed.). University College London. http://www.bslcorpusproject.org/

[bibr57-00238309221107002] Schembri A. Fenlon J. Rentelis R. Reynolds S. Cormier K. (2013). Building the British Sign Language Corpus. Language Documentation & Conservation, 7, 136–154.

[bibr58-00238309221107002] Schermer T. (2001). The role of mouthings in sign language of the Netherlands: Some implications for the production of sign language dictionaries. In Boyes Braem P. Sutton-Spence R. (Eds.), The hands are the head of the mouth: The mouth as articulator in sign languages (pp. 273–284). Signum Press.

[bibr59-00238309221107002] Siu W. R. (2007). Mouth gestures in Hong Kong Sign Language (HKSL): A preliminary study [Unpublished master’s thesis]. The Chinese University of Hong Kong.

[bibr60-00238309221107002] Stamp R. (2013). Sociolinguistic variation, language change and contact in the British Sign Language (BSL) lexicon (Part 1) [Unpublished doctoral thesis]. University College London.

[bibr61-00238309221107002] Stamp R. (2016). Do signers understand regional varieties of a sign language? A lexical recognition experiment. Journal of Deaf Studies and Deaf Education, 21(1), 83–93. 10.1093/deafed/env044 26399952

[bibr62-00238309221107002] Stamp R. Schembri A. Fenlon J. Rentelis R. Woll B. Cormier K. (2014). Lexical variation and change in British Sign Language. PLOS ONE, 9(4), Article e94053. 10.1371/journal.pone.0094053 PMC3997342 24759673

[bibr63-00238309221107002] Sutton-Spence R. (1994). The role of the manual alphabet and fingerspelling in British Sign Language [Unpublished doctoral thesis]. University of Bristol.

[bibr64-00238309221107002] Sutton-Spence R. Day L. (2001). Mouthings and mouth gestures in British Sign Language. In Boyes Braem P. Sutton-Spence R. (Eds.), The hands are the head of the mouth: The mouth as articulator in sign languages (pp. 69–87). Signum Press.

[bibr65-00238309221107002] Sutton-Spence R. Woll B. Allsop L. (1990). Variation and recent change in fingerspelling in British Sign Language. Language Variation and Change, 2(3), 313–330. 10.1017/S0954394500000399

[bibr66-00238309221107002] Van De Sande I. Crasborn O. (2009). Lexically bound mouth actions in Sign Language of the Netherlands: A comparison between different registers and age groups. Linguistics in the Netherlands, 26(1), 78–90. 10.1075/avt.26.08san

[bibr67-00238309221107002] Vinson D. P. Thompson R. L. Skinner R. Fox N. Vigliocco G. (2010). The hands and mouth do not always slip together in British Sign Language: Dissociating articulatory channels in the lexicon. Psychological Science, 21(8), 1158–1167. 10.1177/0956797610377340 20644107

[bibr68-00238309221107002] Vogt-Svendsen M. (2001). A comparison of mouth gestures and mouthings in Norwegian Sign Language (NSL). In Boyes Braem P. Sutton-Spence R. (Eds.), The hands are the head of the mouth: The mouth as articulator in sign languages (pp. 9–40). Signum Press.

[bibr69-00238309221107002] Wei L. (2018a). Translanguaging and code-switching: What’s the difference? OUPblog. https://blog.oup.com/2018/05/translanguaging-code-switching-difference/

[bibr70-00238309221107002] Wei L. (2018b). Translanguaging as a Practical Theory of Language. Applied Linguistics, 39(1), 9–30. 10.1093/applin/amx039

