# Supplementary material for: Sociolinguistic Variation in Mouthings in British Sign Language: A Corpus-Based Study
Source: Lang Speech. 2022 Jul 28;66(2):412–41. doi: 10.1177/00238309221107002 (PMC10230597; doi:10.1177/00238309221107002)
Supplement: sj-html-1-las-10.1177_00238309221107002 – Supplemental material for Sociolinguistic Variation in Mouthings in British Sign Language: A Corpus-Based Study [file sj-html-1-las-10.1177_00238309221107002.html]

Sociolinguistic Variation in Mouthings in BSL


# Sociolinguistic Variation in Mouthings in BSL

#### Heidi Proctor

#### 2022-02-09 14:57:30

# 1 Read in and manipulate the data.

NOTES:

Abbreviations used in comments to this code:

MA = mouth activity

M = mouthing

MG = mouth gesture.

```
library(plyr) # for joins & summary tables for chi square
library(tidyverse) # includes ggplot for visualisations
```

```
## ── Attaching packages ─────────────────────────────────────── tidyverse 1.3.1 ──
```

```
## ✓ ggplot2 3.3.5     ✓ purrr   0.3.4
## ✓ tibble  3.1.6     ✓ dplyr   1.0.7
## ✓ tidyr   1.1.4     ✓ stringr 1.4.0
## ✓ readr   2.1.1     ✓ forcats 0.5.1
```

```
## ── Conflicts ────────────────────────────────────────── tidyverse_conflicts() ──
## x dplyr::arrange()   masks plyr::arrange()
## x purrr::compact()   masks plyr::compact()
## x dplyr::count()     masks plyr::count()
## x dplyr::failwith()  masks plyr::failwith()
## x dplyr::filter()    masks stats::filter()
## x dplyr::id()        masks plyr::id()
## x dplyr::lag()       masks stats::lag()
## x dplyr::mutate()    masks plyr::mutate()
## x dplyr::rename()    masks plyr::rename()
## x dplyr::summarise() masks plyr::summarise()
## x dplyr::summarize() masks plyr::summarize()
```

```
library(lme4) # for mixed effects models
```

```
## Loading required package: Matrix
```

```
## 
## Attaching package: 'Matrix'
```

```
## The following objects are masked from 'package:tidyr':
## 
##     expand, pack, unpack
```

```
library(rcompanion) # for calculating Cramer's V
library(glue) # for concatenating strings for graph elements

knitr::opts_chunk$set(echo = TRUE, warning = FALSE, message= FALSE)
options(qwraps2_markup = "markdown", warn = 1)
```

```
MouthActivityDf <-read.csv(here::here("data", 
  "Mouth-Activity-Raw-Data.csv"), stringsAsFactors = FALSE)

# rename some columns with more meaningful names:
names(MouthActivityDf)[names(MouthActivityDf) 
                       == "Annotation1.2"] <- "idGloss"
names(MouthActivityDf)[names(MouthActivityDf) 
                       == "HitPositionInTier"] <- "idGlossHitPosition"
names(MouthActivityDf)[names(MouthActivityDf) 
                       == "BeginTime"] <- "idGlossBeginTime"
names(MouthActivityDf)[names(MouthActivityDf) 
                       == "TierName"] <- "RhLh"
names(MouthActivityDf)[names(MouthActivityDf) 
                       == "HitPositionInTier.1"] <- "GCHitPosition"
names(MouthActivityDf)[names(MouthActivityDf) 
                       == "BeginTime.1"] <- "GCBeginTime"
names(MouthActivityDf)[names(MouthActivityDf) 
                       == "Annotation3.2"] <- "mouthActivity"
names(MouthActivityDf)[names(MouthActivityDf) 
                       == "HitPositionInTier.2"] <- "MAHitPosition"
names(MouthActivityDf)[names(MouthActivityDf) 
                       == "BeginTime.2"] <- "MABeginTime"
names(MouthActivityDf)[names(MouthActivityDf) 
                       == "X"] <- "verbType"
names(MouthActivityDf)[names(MouthActivityDf) 
                       == "X.1"] <- "North or South"

# Drop unecessary column - this is covered by "verbType":
MouthActivityDf$"Annotation2.2" <- NULL

# Original data had "No/other mouth activity" coded as "None". So change "None" 
# to be "None/Other".  Also expand "MG" to be "Mouth Gesture":
MouthActivityDf[MouthActivityDf == "None"] <- "None/Other"
MouthActivityDf[MouthActivityDf == "MG"] <- "Mouth Gesture"

# Strip the .eaf suffix AND WHETHER THE TEXT TYPE IS CONVERSATION OR NARRATIVE
# from TransriptionName.
# Then rename the column to "participant"
for (i in 1:nrow(MouthActivityDf)) {
  MouthActivityDf[i, "TranscriptionName"] <- 
    str_trunc(MouthActivityDf[i, "TranscriptionName"], 9, ellipsis = "")
}
names(MouthActivityDf)[names(MouthActivityDf) 
                       == "TranscriptionName"] <- "participant"
  
# Add column to identify whether a verb is Plain or not
MouthActivityDf$isPlainVerb <- FALSE
MouthActivityDf$isPlainVerb <- 
  MouthActivityDf$verbType == "PlainVerb"

# Add column to indicate whether participant is from the north (participant 
# starts GW or BF) as opposed to south (participant starts LN or BR)
MouthActivityDf$isNorth <- FALSE
MouthActivityDf$isNorth <- str_detect(MouthActivityDf$participant, "GW") |
  str_detect(MouthActivityDf$participant, "BF")

# Add column to indicate whether each mouth activity is Mouthing or not, to 
# create a binary response variable:
MouthActivityDf$isMouthing <- MouthActivityDf$mouthActivity == "Mouthing"
```

```
# Summarise the data: calculate % of M, MG, None/Other for each participant:

MAByParticipantWide <- 
  aggregate(MouthActivityDf$mouthActivity, 
            by = list(MouthActivityDf$participant, 
                      MouthActivityDf$mouthActivity,
                      MouthActivityDf$isNorth), 
            FUN = length) %>%
  pivot_wider(names_from = Group.2, 
              values_from = x)

names(MAByParticipantWide)[names(MAByParticipantWide) ==
                                    "Group.1"] <- "participant"

names(MAByParticipantWide)[names(MAByParticipantWide) ==
                                    "Group.3"] <- "isNorth"

# Clarify that the values in Mouthing, Mouth Gesture and None/Other columns are
# counts, by renaming the columns:
names(MAByParticipantWide)[names(MAByParticipantWide) ==
                                    "Mouthing"] <- "Mouthing Count"
names(MAByParticipantWide)[names(MAByParticipantWide) ==
                                    "Mouth Gesture"] <- "Mouth Gesture Count"
names(MAByParticipantWide)[names(MAByParticipantWide) ==
                                    "None/Other"] <- "None/Other Count"

# Instances where a participant produces zero M, MG or None/Other are shown as 
# NA.
# Replace these with zeroes
MAByParticipantWide[is.na(MAByParticipantWide)] <- 0

# Add number of tokens per participant
MAByParticipantWide$totalTokens <- 
  MAByParticipantWide$"Mouthing Count" + 
  MAByParticipantWide$"Mouth Gesture Count" + 
  MAByParticipantWide$"None/Other Count"

# Add percentage of tokens that are M, MG or None/Other respectively.
# ("Percentage" is assumed & not included in the variable name so that the graph 
# legends are correct)
MAByParticipantWide$"Mouth Gesture" <- 
  100 * MAByParticipantWide$"Mouth Gesture Count" / 
  MAByParticipantWide$totalTokens
MAByParticipantWide$Mouthing <- 
  100 * MAByParticipantWide$"Mouthing Count" / MAByParticipantWide$totalTokens
MAByParticipantWide$"None/Other" <- 
  100 * MAByParticipantWide$"None/Other Count" / MAByParticipantWide$totalTokens

# Add a ranking based on percentageM, so as to show participants in increasing
# percentage of mouthing in the MA by Participant graph
# First, sort the data by percentageM, then add a sequential rank value 1-100
MAByParticipantWide <- arrange(MAByParticipantWide, Mouthing)
MAByParticipantWide$percentageMRank <- c(1:nrow(MAByParticipantWide))

# Make the data "long" again so that we can graph split by mouthActivity
MAByParticipantLong <- 
  gather(MAByParticipantWide, mouthActivity, percentMouthActivity, 
         "Mouthing", "Mouth Gesture", "None/Other")

# Change mouthActivity from a character variable to a factor variable, & sort it
# for display in Mouth Activity by the various social factors graphs: 
MAByParticipantLong$mouthActivity <- 
  factor(MAByParticipantLong$mouthActivity, 
         levels = c("Mouthing", "Mouth Gesture", "None/Other"))

# Add column northSouth, with values North and South, based on isNorth column.
# This will ensure the graph labels are North and South rather than TRUE &
# FALSE.
MAByParticipantLong <- mutate(MAByParticipantLong, 
                              northSouth = case_when(
                                isNorth == TRUE ~ "North",
                                isNorth == FALSE ~ "South"
                                ))
```

```
# Summarise the data: calculate % of plain/non-plain verbs for each participant:

VerbTypeByParticipantWide <- 
  aggregate(MouthActivityDf$verbType, 
            by = list(MouthActivityDf$participant, 
                      MouthActivityDf$verbType,
                      MouthActivityDf$"North or South"), 
            FUN = length) %>%
  pivot_wider(names_from = Group.2, 
              values_from = x)

names(VerbTypeByParticipantWide)[names(VerbTypeByParticipantWide) ==
                                    "Group.1"] <- "participant"
names(VerbTypeByParticipantWide)[names(VerbTypeByParticipantWide) ==
                                    "Group.3"] <- "NorthOrSouth"
names(VerbTypeByParticipantWide)[names(VerbTypeByParticipantWide) ==
                                    "NonPlainVerb"] <- "NonPlainVerbCount"
names(VerbTypeByParticipantWide)[names(VerbTypeByParticipantWide) ==
                                    "PlainVerb"] <- "PlainVerbCount"

# Instances where a participant produces zero M, MG or None/Other are shown as NA.
# Replace these with zeroes
VerbTypeByParticipantWide[is.na(VerbTypeByParticipantWide)] <- 0

# Add number of tokens per participant, then percentage of tokens that are
# Plain or Non-plain respectively
# (% is assumed & not in the variable name so that the graph legends are 
# correct)
VerbTypeByParticipantWide$totalTokens <- 
  VerbTypeByParticipantWide$NonPlainVerbCount + 
  VerbTypeByParticipantWide$PlainVerbCount

VerbTypeByParticipantWide$"Non Plain Verb" <- 
  100 * VerbTypeByParticipantWide$NonPlainVerbCount / 
  VerbTypeByParticipantWide$totalTokens

VerbTypeByParticipantWide$PlainVerb <- 
  100 * VerbTypeByParticipantWide$PlainVerbCount / 
  VerbTypeByParticipantWide$totalTokens

# Add a ranking based on PlainVerb, so as to show participants in increasing
# percentage of plain verbs in the Verb Type by Participant graph
# First, sort the data by PlainVerb, then add a sequential rank value 1-100
VerbTypeByParticipantWide <- arrange(VerbTypeByParticipantWide, PlainVerb)
VerbTypeByParticipantWide$PlainVerbRank <- c(1:nrow(VerbTypeByParticipantWide))

names(VerbTypeByParticipantWide)[names(VerbTypeByParticipantWide) 
                                 == "PlainVerb"] <- "Plain Verb"
```

```
participantData <-
  read.csv(here::here("data", 
                      "Participant_Data.csv"), 
                      stringsAsFactors = FALSE)
participantData$X<- NULL

# Expand names of cities from their initials to the full names 
participantData[participantData == "BF"] <- "Belfast"
participantData[participantData == "BL"] <- "Bristol"
participantData[participantData == "GW"] <- "Glasgow"
participantData[participantData == "LN"] <- "London"

# Add the participant data to MouthActivityDf, MAByParticipantWide &
# MAByParticipantLong using leftjoin. 

MouthActivityDf <- 
  left_join(MouthActivityDf, participantData, by = "participant")
MAByParticipantWide <- 
  left_join(MAByParticipantWide, participantData, by = "participant")
MAByParticipantLong <- 
  left_join(MAByParticipantLong, participantData, by = "participant")

# Create values of the x-axis of Participant graphs,
# concatenating participantCode & totalTokens
# giving us, for example, "LN19 (10)"

MAByParticipantLong$participantAndTokenCount <- 
  glue("
       {MAByParticipantLong$participantCode}", 
       " ({MAByParticipantLong$totalTokens})
       ")
```

```
# Create df IDGlossMACountWide, to be used to graph MA by verb type, and % of MA 
# for frequently-occurring tokens
IDGlossMACountWide <- 
  aggregate(MouthActivityDf$idGloss, 
            by = list(MouthActivityDf$idGloss,
                      MouthActivityDf$mouthActivity,
                      MouthActivityDf$isPlainVerb),
            FUN = length) %>%
  pivot_wider(names_from = Group.2,
              values_from = x)

# Replace NA values with zero, then calculate the total number of tokens for 
# each ID gloss. Give the idGloss & Verb Type columns a meaningful name.
# Replace isPlainVerb true/false with Plain Verb/Non-Plain Verb
IDGlossMACountWide[is.na(IDGlossMACountWide)] <- 0
IDGlossMACountWide$tokenCount <- 
  IDGlossMACountWide$"Mouth Gesture" + 
  IDGlossMACountWide$Mouthing + 
  IDGlossMACountWide$"None/Other"

names(IDGlossMACountWide)[names(IDGlossMACountWide) ==
                                    "Group.1"] <- "idGloss"
names(IDGlossMACountWide)[names(IDGlossMACountWide) ==
                                    "Group.3"] <- "verbType"

IDGlossMACountWide$verbType <- 
  str_replace(IDGlossMACountWide$verbType, "TRUE", "Plain Verb")
IDGlossMACountWide$verbType <- 
  str_replace(IDGlossMACountWide$verbType, "FALSE", "Non-plain Verb")

# Clarify that the values in Mouthing, Mouth Gesture and None/Other columns are
# counts - rename the columns:
names(IDGlossMACountWide)[names(IDGlossMACountWide) ==
                                    "Mouthing"] <- "Mouthing Count"
names(IDGlossMACountWide)[names(IDGlossMACountWide) ==
                                    "Mouth Gesture"] <- "Mouth Gesture Count"
names(IDGlossMACountWide)[names(IDGlossMACountWide) ==
                                    "None/Other"] <- "None/Other Count"

# Add percentage of tokens that are M, MG or None respectively
# (% is assumed & not in the variable name so that the graph legends are 
# correct)
IDGlossMACountWide$"Mouth Gesture" <- 
  100 * IDGlossMACountWide$"Mouth Gesture Count" / IDGlossMACountWide$tokenCount
IDGlossMACountWide$"Mouthing" <- 
  100 * IDGlossMACountWide$"Mouthing Count" / IDGlossMACountWide$tokenCount
IDGlossMACountWide$"None/Other" <- 
  100 * IDGlossMACountWide$"None/Other Count" / IDGlossMACountWide$tokenCount

# Make the IDGlossMACountWide into Long data so we have separate rows
# for "Mouthing", "Mouth Gesture" & "None/Other", ready to graph
IDGlossMACountLong <- 
  gather(IDGlossMACountWide, mouthActivity, percentMouthActivity, 
         "Mouthing", "Mouth Gesture", "None/Other")

# reorder the values of mouthActivity so that they appear as Mouthing, Mouth
# Gesture then None/Other in the Mouth Activity by Verb Type graph
IDGlossMACountLong$mouthActivity <- 
  factor(IDGlossMACountLong$mouthActivity, 
         levels = c("Mouthing", "Mouth Gesture", "None/Other"))
  
# Create FrequentIDGlossMACountWide, including just those IDglosses which occur 
# more than 9 times: these will be inculded in the MA by Frequent IDGloss graph:
FrequentIDGlossMACountWide <- 
  IDGlossMACountWide[IDGlossMACountWide$tokenCount > 9, ]

# Add a ranking based on percentageM, so as to show IDGlosses in increasing
# percentage of mouthing in the MA by Frequent IDGloss graph
# First, sort the data by (percentage) Mouthing, then add a sequential rank 
# value 1-100
FrequentIDGlossMACountWide <- arrange(FrequentIDGlossMACountWide, Mouthing)
FrequentIDGlossMACountWide$percentageMRank <- 
  c(1:nrow(FrequentIDGlossMACountWide))

# Create FrequentIDGlossMACountLong
FrequentIDGlossMACountLong <- 
  gather(FrequentIDGlossMACountWide, mouthActivity, percentMouthActivity, 
         "Mouthing", "Mouth Gesture", "None/Other")
 
# Create values of the x-axis of the Mouth Activity by Frequent ID Gloss graph,
# concatenating idGloss & totalTokens
FrequentIDGlossMACountLong$IdGlossAndTokenCount <- 
  glue("
       {FrequentIDGlossMACountLong$idGloss}", 
       " ({FrequentIDGlossMACountLong$tokenCount})
       ")

# Change mouthActivity from a character variable to a factor variable, & sort it
# for display in Mouth Activity by (Frequent) IDGloss graph: 
FrequentIDGlossMACountLong$mouthActivity <- 
  factor(FrequentIDGlossMACountLong$mouthActivity, 
         levels = c("None/Other", "Mouth Gesture", "Mouthing"))
```

```
# Create a function that returns the min and max percentages of mouthing,
# mouth gesture and None/Other in the input data frame, split by the input social 
# factor
calcMinMax <- function(df, socialFactor = NULL) {
  minMAByFactor <- aggregate(df$percentMouthActivity,
                             by = list(df$mouthActivity, 
                                       df[[socialFactor]]),
                             FUN = min) 
  
  names(minMAByFactor)[names(minMAByFactor) == "Group.1"] <- "Mouth Activity"
  names(minMAByFactor)[names(minMAByFactor) == "Group.2"] <- socialFactor
  names(minMAByFactor)[names(minMAByFactor) == "x"] <- "Min"
  
  minMAByFactor$Min <- round(minMAByFactor$Min, 0)
  
  maxMAByFactor <- aggregate(df$percentMouthActivity,
                             by = list(df$mouthActivity, 
                                       df[[socialFactor]]),
                             FUN = max) 
  
  names(maxMAByFactor)[names(maxMAByFactor) == "Group.1"] <- "Mouth Activity"
  names(maxMAByFactor)[names(maxMAByFactor) == "Group.2"] <- socialFactor
  names(maxMAByFactor)[names(maxMAByFactor) == "x"] <- "Max"
  
  maxMAByFactor$Max <- round(maxMAByFactor$Max, 0)
  
  minMaxMAByFactor <- join(minMAByFactor, maxMAByFactor)
  
  return(minMaxMAByFactor)
}
```

```
# Set up standard features of all the graphs:
verJustXAxis = -0.25
verJustYAxis = -0.75

theme_set(theme_classic(base_size = 10))
theme_update(axis.title.x = element_text(vjust = verJustXAxis, size = 10),
      axis.title.y = element_text(vjust = verJustYAxis, size = 10),
      axis.text = element_text(size = 9, colour = "black"),
      legend.title = element_blank())
```

# 2 Results

## 2.1 Rate of Mouth Activity, split by Linguistic and Social Factors

### 2.1.1 Mouth Activity Overall

Calculate the min & max percentage of mouthing, mouth gesture and None/Other, overall:

```
minMAOverall <- aggregate(MAByParticipantLong$percentMouthActivity,
                             by = list(MAByParticipantLong$mouthActivity),
                             FUN = min) 

names(minMAOverall)[names(minMAOverall) == "Group.1"] <- "Mouth Activity"
names(minMAOverall)[names(minMAOverall) == "x"] <- "Min"

minMAOverall$Min <- round(minMAOverall$Min, 0)

maxMAOverall <- aggregate(MAByParticipantLong$percentMouthActivity,
                           by = list(MAByParticipantLong$mouthActivity),
                           FUN = max) 

names(maxMAOverall)[names(maxMAOverall) == "Group.1"] <- "Mouth Activity"
names(maxMAOverall)[names(maxMAOverall) == "x"] <- "Max"

maxMAOverall$Max <- round(maxMAOverall$Max, 0)

minMaxMAOverall <- join(minMAOverall, maxMAOverall)
minMaxMAOverall
```

```
ggplot(MAByParticipantLong, aes(x = mouthActivity,
                                y = percentMouthActivity, 
                                fill = mouthActivity,
                                colour = mouthActivity)) +
  geom_boxplot(outlier.colour="black", outlier.shape=16,
             outlier.size = 2, 
             notch = FALSE,
             width = 0.25,
             colour = "Black",
             show.legend = FALSE) +
  scale_fill_brewer(palette = "Greys",
                    direction = -1) +
  scale_y_continuous(expand = c(0, 0)) + 
  labs(x = "Type of mouth activity", y = "Percentage mouth activity")
```

```
ggsave(file = "../results/MA Overall boxplot.eps", width = 6)
```

## 2.2 Mouth Activity by Participant

```
# Re-sort the levels of mouthActivity for display in these graphs, so that
# mouthing appears as the lowest bar, with MG above then "None/Other" at the top: 
MAByParticipantLong$mouthActivity <- 
  factor(MAByParticipantLong$mouthActivity, 
         levels = c("None/Other", "Mouth Gesture", "Mouthing"))

# In the graph, sort the data by percentageMRank so the participants are ordered 
# from least M to most M:
ggplot(MAByParticipantLong, aes(x = reorder(participantAndTokenCount, 
                                            percentageMRank), 
                                y = percentMouthActivity, 
                                fill = mouthActivity
                                ,
                                order = desc(mouthActivity))) +
  geom_bar(stat="identity") +
  scale_fill_brewer(palette = "Greys",
                  guide=guide_legend(reverse=T)) +
  theme(axis.text.x = element_text(angle = 90,
                                   hjust = 1, 
                                   size = 6), 
        legend.position = "bottom") +
  scale_y_continuous(expand = c(0, 0)) +
  labs(x = "Participant (number of tokens)", y = "Percentage mouth activity")
```

```
ggsave(file = "../results/MA by Participant.eps")

# Reset the sorting of mouthActivity so it is correct for all graphs split by
# social factors, which also use this df: 
MAByParticipantLong$mouthActivity <- 
  factor(MAByParticipantLong$mouthActivity, 
         levels = c("Mouthing", "Mouth Gesture", "None/Other"))
```

## 2.3 Mouth Activity by (Frequent) IDGloss

```
# In the graph, sort the data by percentageMRank so it is ordered from least M
# to most M:
ggplot(FrequentIDGlossMACountLong, aes(x = reorder(IdGlossAndTokenCount, 
                                                   percentageMRank),
                                y = percentMouthActivity, 
                                fill = mouthActivity)) +
  geom_bar(stat="identity") +
  scale_fill_brewer(palette = "Greys",
                    guide = guide_legend(reverse = TRUE)) +
  theme(axis.text.x = element_text(angle = 90,
                                   hjust = 1),
        legend.position = "bottom",
        aspect.ratio = 1/2.5,
        plot.margin = unit(c(0.3, 0.1, 0, 0.1), "cm")) +
#  coord_fixed(ratio = 0.2) +
  scale_y_continuous(expand = c(0, 0)) +
  labs(x = "Verb (number of tokens)", y = "Percentage mouth activity")
```

```
ggsave(file = "../results/MA by ID Gloss.eps")
```

### 2.3.1 Mouth Activity Split by Verb Type

Calculate Chi-Square:

```
# Create a cross-tab contingency table for plain/non-plain verbs, and 
# mouthing/not mouthing
VerbTypeCrossTab <- xtabs(formula = ~ isPlainVerb + isMouthing, 
                          data = MouthActivityDf)
VerbTypeCrossTab
```

```
##            isMouthing
## isPlainVerb FALSE TRUE
##       FALSE   519  761
##       TRUE    125  366
```

```
# Chi-Squared test:
chisq.test(MouthActivityDf$isPlainVerb, MouthActivityDf$isMouthing, 
           correct = FALSE)
```

```
## 
##  Pearson's Chi-squared test
## 
## data:  MouthActivityDf$isPlainVerb and MouthActivityDf$isMouthing
## X-squared = 34.914, df = 1, p-value = 3.446e-09
```

```
# Next - add Cramer's V calculation
cramerV(VerbTypeCrossTab)
```

```
## Cramer V 
##   0.1404
```

Calculate the min & max percentage of mouthing, mouth gesture and None/Other, split by verb type:

```
minMAByVerbType <- aggregate(IDGlossMACountLong$percentMouthActivity,
                             by = list(IDGlossMACountLong$mouthActivity, 
                                       IDGlossMACountLong[["verbType"]]),
                             FUN = min) 

names(minMAByVerbType)[names(minMAByVerbType) == "Group.1"] <- "Mouth Activity"
names(minMAByVerbType)[names(minMAByVerbType) == "Group.2"] <- "Verb Type"
names(minMAByVerbType)[names(minMAByVerbType) == "x"] <- "Min"

minMAByVerbType$Min <- round(minMAByVerbType$Min, 0)

maxMAByVerbType <- aggregate(IDGlossMACountLong$percentMouthActivity,
                           by = list(IDGlossMACountLong$mouthActivity, 
                                       IDGlossMACountLong[["verbType"]]),
                           FUN = max) 

names(maxMAByVerbType)[names(maxMAByVerbType) == "Group.1"] <- "Mouth Activity"
names(maxMAByVerbType)[names(maxMAByVerbType) == "Group.2"] <- "Verb Type"
names(maxMAByVerbType)[names(maxMAByVerbType) == "x"] <- "Max"

maxMAByVerbType$Max <- round(maxMAByVerbType$Max, 0)

minmaxMAByVerbType <- join(minMAByVerbType, maxMAByVerbType)
minmaxMAByVerbType
```

```
ggplot(IDGlossMACountLong, aes(x = verbType,
                               y = percentMouthActivity,  
                               fill = mouthActivity,
                               colour = mouthActivity)) +
  geom_boxplot(outlier.colour="black", 
               outlier.shape=16,
               outlier.size=2, 
               notch=FALSE,
               colour = "Black") +
  scale_fill_brewer(palette = "Greys",
                    direction = -1) +
  scale_y_continuous(expand = c(0, 0)) +
  labs(x = "Verb type", y = "Percentage mouth activity")
```

```
ggsave(file = "../results/MA by Verb Type boxplot.eps")
```

### 2.3.2 Mouth Activity Split by City

Calculate Chi-Square:

```
# Create a cross-tab contingency table for the 4 cities, and 
# mouthing/not mouthing:
CityCrossTab <- xtabs(formula = ~ region + isMouthing,  data = MouthActivityDf)
CityCrossTab
```

```
##          isMouthing
## region    FALSE TRUE
##   Belfast   169  244
##   Bristol   126  301
##   Glasgow   180  260
##   London    169  322
```

```
# Chi-Squared test:
chisq.test(MouthActivityDf$region, MouthActivityDf$isMouthing,
           correct = FALSE)
```

```
## 
##  Pearson's Chi-squared test
## 
## data:  MouthActivityDf$region and MouthActivityDf$isMouthing
## X-squared = 17.108, df = 3, p-value = 0.0006715
```

```
# Next - add Cramer's V calculation
cramerV(CityCrossTab)
```

```
## Cramer V 
##  0.09829
```

Calculate the min & max percentage of mouthing, mouth gesture and None/Other, split by city:

```
calcMinMax(MAByParticipantLong, "region")
```

```
# Re-order the cities so that the 2 North cities are first followed by the 2
# South cities:
MAByParticipantLong <- mutate(MAByParticipantLong, 
                              region = factor(region, 
                                              levels = c("Belfast", "Glasgow",
                                                         "Bristol", "London")))

ggplot(MAByParticipantLong, aes(x = region,
                                y = percentMouthActivity, 
                                fill = mouthActivity)) +
  geom_boxplot(outlier.colour="black", outlier.shape=16,
             outlier.size=2, notch=FALSE,
             ) +
  scale_fill_brewer(palette = "Greys",
                    direction = -1) +
  scale_y_continuous(expand = c(0, 0)) + 
  labs(x = "City", y = "Percentage mouth activity")
```

```
ggsave(file = "../results/MA by City boxplot.eps")
```

### 2.3.3 Mouth Activity Split by North/South

Calculate Chi-Square:

```
# Create a cross-tab contingency table for North versus South, and 
# mouthing/not mouthing:
NorthSouthCrossTab <- xtabs(formula = ~ isNorth + isMouthing,  
                            data = MouthActivityDf)
NorthSouthCrossTab
```

```
##        isMouthing
## isNorth FALSE TRUE
##   FALSE   295  623
##   TRUE    349  504
```

```
# Chi-Squared test:
chisq.test(MouthActivityDf$isNorth, MouthActivityDf$isMouthing, correct = FALSE)
```

```
## 
##  Pearson's Chi-squared test
## 
## data:  MouthActivityDf$isNorth and MouthActivityDf$isMouthing
## X-squared = 14.727, df = 1, p-value = 0.0001242
```

```
# Next - add Cramer's V calculation
cramerV(NorthSouthCrossTab)
```

```
## Cramer V 
##  0.09119
```

Calculate the min & max percentage of mouthing, mouth gesture and None/Other, split by north/south:

```
calcMinMax(MAByParticipantLong, "northSouth")
```

```
ggplot(MAByParticipantLong, aes(x = northSouth,
                                y = percentMouthActivity, 
                                fill = mouthActivity)) +
  geom_boxplot(outlier.colour="black", outlier.shape=16,
             outlier.size=2, notch=FALSE) +
  scale_fill_brewer(palette = "Greys",
                    direction = -1) +
  scale_y_continuous(expand = c(0, 0)) +
  labs(x = "Region", y = "Percentage mouth activity")
```

```
ggsave(file = "../results/MA by North-South boxplot.eps")
```

### 2.3.4 Mouth Activity Split by Gender

Calculate Chi-Square:

```
# Create a cross-tab contingency table for gender, and 
# mouthing/not mouthing:
GenderCrossTab <- xtabs(formula = ~ gender + isMouthing, 
                        data = MouthActivityDf)
GenderCrossTab
```

```
##         isMouthing
## gender   FALSE TRUE
##   Female   319  638
##   Male     325  489
```

```
# Chi-Squared test:
chisq.test(MouthActivityDf$gender, MouthActivityDf$isMouthing, 
           correct = FALSE)
```

```
## 
##  Pearson's Chi-squared test
## 
## data:  MouthActivityDf$gender and MouthActivityDf$isMouthing
## X-squared = 8.2624, df = 1, p-value = 0.004047
```

```
# Next - add Cramer's V calculation
cramerV(GenderCrossTab)
```

```
## Cramer V 
##   0.0683
```

```
calcMinMax(MAByParticipantLong, "gender")
```

```
ggplot(MAByParticipantLong, aes(x = gender, 
                                y = percentMouthActivity,
                                fill = mouthActivity)) +
  geom_boxplot(outlier.colour="black", outlier.shape=16,
             outlier.size=2, notch=FALSE) +
  scale_fill_brewer(palette = "Greys",
                    direction = -1) +
  scale_y_continuous(expand = c(0, 0)) +
  labs(x = "Gender", y = "Percentage mouth activity")
```

```
ggsave(file = "../results/MA by Gender boxplot.eps")
```

### 2.3.5 Mouth Activity Split by Age Group

Calculate Chi-Square:

```
# First - investigate Age = Mid versus Age <> Mid, since our hypothesis is that
# those where Age = Mid will produce more mourthing than the other groups.
MouthActivityDf$isAgeMid <- MouthActivityDf$age4Cats == "M"

# Create a cross-tab contingency table for Age-Mid versus Age-not-Mid, and 
# mouthing/not mouthing:
isAgeMidCrossTab <- xtabs(formula = ~ isAgeMid + isMouthing,  
                             data = MouthActivityDf)
isAgeMidCrossTab
```

```
##         isMouthing
## isAgeMid FALSE TRUE
##    FALSE   438  758
##    TRUE    206  369
```

```
# Chi-Squared test:
chisq.test(MouthActivityDf$isAgeMid, MouthActivityDf$isMouthing, 
           correct = FALSE)
```

```
## 
##  Pearson's Chi-squared test
## 
## data:  MouthActivityDf$isAgeMid and MouthActivityDf$isMouthing
## X-squared = 0.10632, df = 1, p-value = 0.7444
```

```
# Next - add Cramer's V calculation
cramerV(isAgeMidCrossTab)
```

```
## Cramer V 
## 0.007748
```

```
# This shows that there is no significant difference between the rate of 
# mouthing in those aged 36-50 compared to those in other age groups.

# Next, produce statistics for all 4 age categories:

# Create a cross-tab contingency table for all 4 age categories, and 
# mouthing/not mouthing:
Age4CatsCrossTab <- xtabs(formula = ~ age4Cats + isMouthing,  
                             data = MouthActivityDf)
Age4CatsCrossTab
```

```
##         isMouthing
## age4Cats FALSE TRUE
##        E   115  240
##        M   206  369
##        O   143  269
##        Y   180  249
```

```
# Chi-Squared test:
chisq.test(MouthActivityDf$age4Cats, MouthActivityDf$isMouthing, 
           correct = FALSE)
```

```
## 
##  Pearson's Chi-squared test
## 
## data:  MouthActivityDf$age4Cats and MouthActivityDf$isMouthing
## X-squared = 8.7786, df = 3, p-value = 0.03238
```

```
# Next - add Cramer's V calculation
cramerV(Age4CatsCrossTab)
```

```
## Cramer V 
##   0.0704
```

```
# Unxpectedly, the chi-squared statistic for Age4Cats is significant.
# 
# Look at the square of the residuals in the chi-square calculation to 
# determine which age group(s) are contributing to the significant difference: 

chisq.test(MouthActivityDf$age4Cats, MouthActivityDf$isMouthing, 
           correct = FALSE)$residuals^2 %>% round(digits = 2)
```

```
##                         MouthActivityDf$isMouthing
## MouthActivityDf$age4Cats FALSE TRUE
##                        E  1.54 0.88
##                        M  0.05 0.03
##                        O  0.31 0.18
##                        Y  3.69 2.11
```

```
# This shows that the largest values are for the Young age group, i.e. it is
# this age group that is driving the difference in rate of mouthing.
# 
# To determine the direction of the difference (i.e. are the Young participants
# producing more, or producing less mouthing than the others?), compare the
# observed figures to the expected figures:

chisq.test(MouthActivityDf$age4Cats, MouthActivityDf$isMouthing, 
           correct = FALSE)$observed
```

```
##                         MouthActivityDf$isMouthing
## MouthActivityDf$age4Cats FALSE TRUE
##                        E   115  240
##                        M   206  369
##                        O   143  269
##                        Y   180  249
```

```
chisq.test(MouthActivityDf$age4Cats, MouthActivityDf$isMouthing, 
           correct = FALSE)$expected %>% round(digits = 0)
```

```
##                         MouthActivityDf$isMouthing
## MouthActivityDf$age4Cats FALSE TRUE
##                        E   129  226
##                        M   209  366
##                        O   150  262
##                        Y   156  273
```

```
# This shows that the Young participants are producing LESS mouthing 
# (249 tokens) than expected (273 tokens).

# so we compare Age = Y versus Age <> Y:
MouthActivityDf$isAgeYoung <- MouthActivityDf$age4Cats == "Y"

# Create a cross-tab contingency table for Age-Young versus Age-not-Young, and 
# mouthing/not mouthing:
isAgeYoungCrossTab <- xtabs(formula = ~ isAgeYoung + isMouthing,  
                             data = MouthActivityDf)
isAgeYoungCrossTab
```

```
##           isMouthing
## isAgeYoung FALSE TRUE
##      FALSE   464  878
##      TRUE    180  249
```

```
# Chi-Squared test:
chisq.test(MouthActivityDf$isAgeYoung, MouthActivityDf$isMouthing, 
           correct = FALSE)
```

```
## 
##  Pearson's Chi-squared test
## 
## data:  MouthActivityDf$isAgeYoung and MouthActivityDf$isMouthing
## X-squared = 7.657, df = 1, p-value = 0.005655
```

```
# Next - add Cramer's V calculation
cramerV(isAgeYoungCrossTab)
```

```
## Cramer V 
##  0.06575
```

```
# This shows that there is a significant difference between the amount of 
# mouthing produced by Young signers compared to those in other age groups.
```

```
calcMinMax(MAByParticipantLong, "age4Cats")
```

```
# graph Age4Cats:
# first, add a column "age4CatsYears" to contain the age ranges e.g. 16-35 
# rather than the codes e.g. Y (=young) - these will be displayed on the x-axis.

MAByParticipantLong <- MAByParticipantLong %>% 
  mutate(age4CatsYears = case_when(age4Cats == "Y" ~ "16-35",
                                   age4Cats == "M" ~ "36-50",
                                   age4Cats == "O" ~ "51-64",
                                   age4Cats == "E" ~ "65-88")) 

ggplot(MAByParticipantLong, aes(x = age4CatsYears, 
                                y = percentMouthActivity,
                                fill = mouthActivity)) +
  geom_boxplot(outlier.colour="black", outlier.shape=16,
             outlier.size=2, notch=FALSE) +
  scale_fill_brewer(palette = "Greys",
                    direction = -1) +
  scale_y_continuous(expand = c(0, 0)) +
  labs(x = "Age (years)", y = "Percentage mouth activity")
```

```
ggsave(file = "../results/MA by Age4Cats boxplot.eps")
```

### 2.3.6 Mouth Activity Split by Language Background

Calculate Chi-Square:

```
# Create a cross-tab contingency table for LB, and mouthing/not mouthing:
LBCrossTab <- xtabs(formula = ~ LB + isMouthing,  data = MouthActivityDf)
LBCrossTab
```

```
##    isMouthing
## LB  FALSE TRUE
##   D   253  467
##   H   391  660
```

```
# Chi-Squared test:
chisq.test(MouthActivityDf$LB, MouthActivityDf$isMouthing, 
           correct = FALSE)
```

```
## 
##  Pearson's Chi-squared test
## 
## data:  MouthActivityDf$LB and MouthActivityDf$isMouthing
## X-squared = 0.78645, df = 1, p-value = 0.3752
```

```
# Next - add Cramer's V calculation
cramerV(LBCrossTab)
```

```
## Cramer V 
##  0.02107
```

```
# This shows that there is no significant difference in the rate of mouthing
# produced by participants with LB = deaf compared to LB = hearing.
```

```
calcMinMax(MAByParticipantLong, "LB")
```

## 2.4 Inferential Statistics

### 2.4.1 Effect of Verb Type (Plain/non-Plain) on Mouthing

```
VerbTypeModelGlmer <- 
  glmer(isMouthing ~ verbType + (1 | participant), 
        data = MouthActivityDf, family = "binomial")
summary(VerbTypeModelGlmer)
```

```
## Generalized linear mixed model fit by maximum likelihood (Laplace
##   Approximation) [glmerMod]
##  Family: binomial  ( logit )
## Formula: isMouthing ~ verbType + (1 | participant)
##    Data: MouthActivityDf
## 
##      AIC      BIC   logLik deviance df.resid 
##   2260.6   2277.1  -1127.3   2254.6     1768 
## 
## Scaled residuals: 
##     Min      1Q  Median      3Q     Max 
## -2.4666 -1.1068  0.5768  0.7564  1.3390 
## 
## Random effects:
##  Groups      Name        Variance Std.Dev.
##  participant (Intercept) 0.2924   0.5407  
## Number of obs: 1771, groups:  participant, 100
## 
## Fixed effects:
##                   Estimate Std. Error z value Pr(>|z|)    
## (Intercept)        0.39328    0.08259   4.762 1.92e-06 ***
## verbTypePlainVerb  0.70516    0.12451   5.664 1.48e-08 ***
## ---
## Signif. codes:  0 '***' 0.001 '**' 0.01 '*' 0.05 '.' 0.1 ' ' 1
## 
## Correlation of Fixed Effects:
##             (Intr)
## vrbTypPlnVr -0.362
```

```
# take the exponential of the fixed effects. this is the log odds:
exp(fixef(VerbTypeModelGlmer))
```

```
##       (Intercept) verbTypePlainVerb 
##          1.481828          2.024163
```

### 2.4.2 Effect of North/South and Gender and Age-Young versus other on Mouthing

This age group was selected because a chi-squared analysis of all 4 age categories revealed that the youngest age group produces less mouthing than the others.

```
NorthSouthGenderAgeYngModelGlmer <- 
  glmer(isMouthing ~ isNorth + gender + isAgeYoung + (1 | participant), 
        data = MouthActivityDf, family = "binomial")
summary(NorthSouthGenderAgeYngModelGlmer)
```

```
## Generalized linear mixed model fit by maximum likelihood (Laplace
##   Approximation) [glmerMod]
##  Family: binomial  ( logit )
## Formula: isMouthing ~ isNorth + gender + isAgeYoung + (1 | participant)
##    Data: MouthActivityDf
## 
##      AIC      BIC   logLik deviance df.resid 
##   2285.7   2313.1  -1137.8   2275.7     1766 
## 
## Scaled residuals: 
##     Min      1Q  Median      3Q     Max 
## -2.1078 -1.1197  0.6115  0.7320  1.3011 
## 
## Random effects:
##  Groups      Name        Variance Std.Dev.
##  participant (Intercept) 0.2358   0.4856  
## Number of obs: 1771, groups:  participant, 100
## 
## Fixed effects:
##                Estimate Std. Error z value Pr(>|z|)    
## (Intercept)      0.9473     0.1279   7.407 1.29e-13 ***
## isNorthTRUE     -0.3660     0.1459  -2.509   0.0121 *  
## genderMale      -0.2766     0.1476  -1.873   0.0610 .  
## isAgeYoungTRUE  -0.2050     0.1688  -1.214   0.2248    
## ---
## Signif. codes:  0 '***' 0.001 '**' 0.01 '*' 0.05 '.' 0.1 ' ' 1
## 
## Correlation of Fixed Effects:
##             (Intr) iNTRUE gndrMl
## isNorthTRUE -0.564              
## genderMale  -0.505  0.019       
## isAgYngTRUE -0.175 -0.111 -0.190
```

```
# take the exponential of the fixed effects. this is the log odds:
exp(fixef(NorthSouthGenderAgeYngModelGlmer))
```

```
##    (Intercept)    isNorthTRUE     genderMale isAgeYoungTRUE 
##      2.5787568      0.6935170      0.7583579      0.8146788
```

```
#coef(NorthSouthGenderAgeYngModelGlmer)
```

# 3 Discussion

## 3.1 Overall rate of MA with Verbs

Rate of Mouthing = 64%

Rate of MG = 19%

Rate of None/Other = 17%

## 3.2 Verb Type

```
# report the percentage of mouthing, MG and "None/Other" for both Plain and
# non-Plain verbs
# 
# (In order for ddply() to work, I remove space from within "Mouth Gesture", 
# then I revert by restoring the space.)
# 
MouthActivityDf[MouthActivityDf == "Mouth Gesture"] <- "MouthGesture"
ddply(MouthActivityDf, 
      "isPlainVerb", 
      summarise, 
     "% Mouthing" = round(mean(mouthActivity == "Mouthing") * 100, 0), 
     "% Mouth Gesture" = round(mean(mouthActivity == "MouthGesture")
                                   * 100, 0), 
     "% None/Other" = round(mean(mouthActivity == "None/Other") * 100, 0))
```

```
MouthActivityDf[MouthActivityDf == "MouthGesture"] <- "Mouth Gesture"
```

## 3.3 City

Report the percentage of mouthing, MG and “None/Other” for each city:

```
MouthActivityDf[MouthActivityDf == "Mouth Gesture"] <- "MouthGesture"
ddply(MouthActivityDf, 
      "region", 
      summarise, 
      "% Mouthing" = round(mean(mouthActivity == "Mouthing") * 100, 0), 
      "% Mouth Gesture" = round(mean(mouthActivity == "MouthGesture")
                                   * 100, 0), 
      "% None/Other" = round(mean(mouthActivity == "None/Other") * 100, 0))
```

```
MouthActivityDf[MouthActivityDf == "MouthGesture"] <- "Mouth Gesture"
```

## 3.4 North/South

Report the percentage of mouthing, MG and “None/Other” for North & South:

```
MouthActivityDf[MouthActivityDf == "Mouth Gesture"] <- "MouthGesture"
ddply(MouthActivityDf, 
      "isNorth", 
      summarise, 
      "% Mouthing" = round(mean(mouthActivity == "Mouthing") * 100, 0), 
      "% Mouth Gesture" = round(mean(mouthActivity == "MouthGesture")
                                   * 100, 0), 
      "% None/Other" = round(mean(mouthActivity == "None/Other") * 100, 0))
```

```
MouthActivityDf[MouthActivityDf == "MouthGesture"] <- "Mouth Gesture"
```

## 3.5 Gender

Report the percentage of mouthing, MG and “% None/Other” for each gender:

```
MouthActivityDf[MouthActivityDf == "Mouth Gesture"] <- "MouthGesture"
ddply(MouthActivityDf, 
      "gender", 
      summarise, 
      "% Mouthing" = round(mean(mouthActivity == "Mouthing") * 100, 0), 
      "% Mouth Gesture" = round(mean(mouthActivity == "MouthGesture")
                                   * 100, 0), 
      "% None/Other" = round(mean(mouthActivity == "None/Other") * 100, 0))
```

```
MouthActivityDf[MouthActivityDf == "MouthGesture"] <- "Mouth Gesture"
```

## 3.6 Age Group

Report the percentage of mouthing, MG and “None/Other” for Age = Mid and Age <> Mid (not significant) Then do the same for Age = Young and Age <> Young (IS significant)

```
MouthActivityDf[MouthActivityDf == "Mouth Gesture"] <- "MouthGesture"

ddply(MouthActivityDf, 
      "isAgeMid", 
      summarise, 
      "% Mouthing" = round(mean(mouthActivity == "Mouthing") * 100, 0), 
      "% Mouth Gesture" = round(mean(mouthActivity == "MouthGesture")
                                   * 100, 0), 
      "% None/Other" = round(mean(mouthActivity == "None/Other") * 100, 0))
```

```
ddply(MouthActivityDf, 
      "isAgeYoung", 
      summarise, 
      "% Mouthing" = round(mean(mouthActivity == "Mouthing") * 100, 0), 
      "% Mouth Gesture" = round(mean(mouthActivity == "MouthGesture")
                                   * 100, 0), 
      "% None/Other" = round(mean(mouthActivity == "None/Other") * 100, 0))
```

```
MouthActivityDf[MouthActivityDf == "MouthGesture"] <- "Mouth Gesture"
```

Report the percentage of mouthing, MG and “None/Other” for LB = deaf and LB = hearing:

```
MouthActivityDf[MouthActivityDf == "Mouth Gesture"] <- "MouthGesture"
ddply(MouthActivityDf, 
      "LB", 
      summarise, 
      "% Mouthing" = round(mean(mouthActivity == "Mouthing") * 100, 0), 
      "% Mouth Gesture" = round(mean(mouthActivity == "MouthGesture")
                                   * 100, 0), 
      "None/Other" = round(mean(mouthActivity == "None/Other") * 100, 0))
```

```
MouthActivityDf[MouthActivityDf == "MouthGesture"] <- "Mouth Gesture"
```

# 4 Session Info

```
sessionInfo()
```

```
## R version 4.1.2 (2021-11-01)
## Platform: x86_64-apple-darwin17.0 (64-bit)
## Running under: macOS Big Sur 10.16
## 
## Matrix products: default
## BLAS:   /Library/Frameworks/R.framework/Versions/4.1/Resources/lib/libRblas.0.dylib
## LAPACK: /Library/Frameworks/R.framework/Versions/4.1/Resources/lib/libRlapack.dylib
## 
## locale:
## [1] en_GB.UTF-8/en_GB.UTF-8/en_GB.UTF-8/C/en_GB.UTF-8/en_GB.UTF-8
## 
## attached base packages:
## [1] stats     graphics  grDevices utils     datasets  methods   base     
## 
## other attached packages:
##  [1] glue_1.6.0       rcompanion_2.4.6 lme4_1.1-27.1    Matrix_1.3-4    
##  [5] forcats_0.5.1    stringr_1.4.0    dplyr_1.0.7      purrr_0.3.4     
##  [9] readr_2.1.1      tidyr_1.1.4      tibble_3.1.6     ggplot2_3.3.5   
## [13] tidyverse_1.3.1  plyr_1.8.6      
## 
## loaded via a namespace (and not attached):
##  [1] nlme_3.1-153       matrixStats_0.61.0 fs_1.5.2           lubridate_1.8.0   
##  [5] RColorBrewer_1.1-2 httr_1.4.2         rprojroot_2.0.2    tools_4.1.2       
##  [9] backports_1.4.1    utf8_1.2.2         R6_2.5.1           nortest_1.0-4     
## [13] DBI_1.1.2          colorspace_2.0-2   withr_2.4.3        tidyselect_1.1.1  
## [17] Exact_3.1          compiler_4.1.2     cli_3.1.1          rvest_1.0.2       
## [21] expm_0.999-6       xml2_1.3.3         sandwich_3.0-1     labeling_0.4.2    
## [25] scales_1.1.1       lmtest_0.9-39      mvtnorm_1.1-3      proxy_0.4-26      
## [29] multcompView_0.1-8 digest_0.6.29      minqa_1.2.4        rmarkdown_2.11    
## [33] pkgconfig_2.0.3    htmltools_0.5.2    highr_0.9          dbplyr_2.1.1      
## [37] fastmap_1.1.0      rlang_0.4.12       readxl_1.3.1       rstudioapi_0.13   
## [41] farver_2.1.0       jquerylib_0.1.4    generics_0.1.1     zoo_1.8-9         
## [45] jsonlite_1.7.2     magrittr_2.0.1     modeltools_0.2-23  Rcpp_1.0.7        
## [49] DescTools_0.99.44  munsell_0.5.0      fansi_0.5.0        lifecycle_1.0.1   
## [53] multcomp_1.4-17    stringi_1.7.6      yaml_2.2.1         MASS_7.3-54       
## [57] rootSolve_1.8.2.3  grid_4.1.2         parallel_4.1.2     crayon_1.4.2      
## [61] lmom_2.8           lattice_0.20-45    haven_2.4.3        splines_4.1.2     
## [65] hms_1.1.1          knitr_1.37         pillar_1.6.4       boot_1.3-28       
## [69] gld_2.6.4          codetools_0.2-18   stats4_4.1.2       reprex_2.0.1      
## [73] evaluate_0.14      data.table_1.14.2  modelr_0.1.8       vctrs_0.3.8       
## [77] nloptr_1.2.2.3     tzdb_0.2.0         cellranger_1.1.0   gtable_0.3.0      
## [81] assertthat_0.2.1   xfun_0.29          coin_1.4-2         libcoin_1.0-9     
## [85] broom_0.7.12       e1071_1.7-9        class_7.3-19       survival_3.2-13   
## [89] TH.data_1.1-0      ellipsis_0.3.2     here_1.0.1
```
